# Demystifying metabolic‒immune crosstalk: how amino acid metabolic reprogramming shapes the malignant phenotype and macrophage polarization of biliary and pancreatic tumors

**DOI:** 10.7150/ijbs.122325

**Published:** 2025-10-27

**Authors:** Jinglei Zhang, Zhuohuan Chu, Jiawen Li, Lu Xie, Cong Ding, Zihui An, Xiang Wang, Hangbin Jin, Xiaofeng Zhang, Qiang Liu, Jianfeng Yang

**Affiliations:** 1 The Fourth School of Clinical Medicine, Zhejiang Chinese Medical University, Hangzhou First People's Hospital, Hangzhou, Zhejiang Province 310053, China.; 2 Department of Gastroenterology, Affiliated Hangzhou First People's Hospital, Westlake University School of Medicine, Hangzhou, Zhejiang Province 310006, China.; 3 Key Laboratory of Integrated Traditional Chinese and Western Medicine for Biliary and Pancreatic Diseases of Zhejiang Province, Hangzhou, Zhejiang Province 310006, China.; 4 Hangzhou Institute of Digestive Diseases, Hangzhou, Zhejiang Province 310006, China.; 5 Hangzhou Cancer Hospital, Hangzhou, Zhejiang Province 310006, China.

**Keywords:** cholangiocarcinoma, pancreatic cancer, amino acid metabolism, metabolic reprogramming, tumor-associated macrophages

## Abstract

Biliary and pancreatic malignant tumors refer to biliary tract carcinoma (BTC) and pancreatic cancer (PC), among which BTC mainly includes cholangiocarcinoma (CCA) and gallbladder cancer (GBC), and their prognosis is poor because of the lack of effective early diagnostic methods. Although surgical resection is the preferred method for a cure, treatment options are limited for patients with advanced tumors. Therefore, the exploration of other new treatment methods is urgently needed. Currently, metabolic reprogramming is a key mechanism in the process of tumor development and progression and is closely related to cancer cell proliferation, metastasis and drug resistance. As an indispensable part of metabolic reprogramming in tumor cells, amino acid (AA) metabolic reprogramming provides an energy source for tumor cells and participates in regulating the tumor microenvironment (TME). Moreover, as important intrinsic myeloid cells, macrophages play indispensable physiological roles in malignant tumor progression. In the TME, tumor cells can not only induce peripheral immune tolerance by releasing extracellular signals but also compete with tumor-associated macrophages (TAMs) for AAs and release the resulting downstream metabolites into the TME, directly targeting and damaging immune cells and influencing macrophage polarization. Consequently, a more profound understanding of the function of AA metabolic reprogramming in biliopancreatic malignancies and their associated macrophage polarization holds the potential to facilitate the development of effective strategies for early diagnosis, prognostic assessment and targeted therapy in patients with biliopancreatic malignancies. In this paper, we review the impact of AA metabolic reprogramming on the occurrence and development of biliary and pancreatic malignant tumors, summarize the relevant mechanisms of AA metabolic reprogramming on the polarization of TAMs, and provide new therapeutic targets for AA metabolic therapies and immunotherapies for biliary and pancreatic malignant tumors.

## 1. Background

In recent years, with improvements in living standards, the incidence and mortality of biliary and pancreatic malignant tumors, primarily CCA, GBC and PC, have increased. Biliary and pancreatic malignant tumors are known to exhibit significant heterogeneity, and most patients typically present with no specific clinical manifestations in the early stages of the disease. Concurrently, owing to the absence of efficacious early screening instruments, the majority of patients are frequently diagnosed at an advanced stage of the disease, with a high degree of malignancy and mortality^1^,^2^. At present, surgical resection remains the preferred treatment for patients with biliary and pancreatic malignant tumors. However, patients have an extremely high rate of recurrence after surgery, and the overall prognosis is poor. The 5-year survival rate for patients with advanced disease is less than 15%, and the median survival time is less than 1 year^3^-^5^. Moreover, for patients without surgical indications, although chemotherapy and radiotherapy are the main treatment options, there are still many challenges, such as high levels of adverse effects, drug resistance and individual differences. Therefore, actively exploring the pathogenesis of biliary and pancreatic malignant tumors and designing scientific diagnostic and therapeutic protocols are highly important.

The TME is defined as the environment surrounding a tumor, which is predominantly comprised of stromal components and immune cells. During the processes of tumorigenesis and progression, a variety of immune cells, including TAMs, dendritic cells (DCs), neutrophils, B cells, T cells and cancer-associated fibroblasts (CAFs), are recruited into the microenvironment surrounding tumor cells. Tumor cells have the capacity to influence the TME by releasing extracellular signals that induce peripheral immune tolerance, while immune cells within the TME can subsequently regulate tumor cell proliferation^6^. TAMs represent a significant component of tumor-infiltrating immune cells within the TME. These cells exhibit a high degree of plasticity, thereby playing a pivotal role in the processes of tumorigenesis and progression^7^. In response to diverse signaling stimuli, TAMs can be polarized into two distinct functional states: classically activated M1-like TAMs and alternatively activated M2-like TAMs. These distinct states are characterized by the expression of specific genes, resulting in a range of biological functions. The polarization of TAMs is a continuous and dynamically changing biological process. In the initial phase of tumor development, M1-like TAMs predominate. These cells function in a multifaceted manner, involving the activation of immune responses, the suppression of tumor cell proliferation, and the induction of chronic inflammation within the organism. This process contributes to the attenuation of tumor cell genome stability^8^. With the progression of tumors to malignancy, the TME, characterized by hypoxia, gradually induces the conversion of M1-like TAMs to M2-like TAMs through direct effects, metabolic regulation, angiogenesis and matrix remodeling mechanisms. M2-like TAMs are associated with immune response suppression and can further promote tumor progression^9^. Recent studies have demonstrated that metabolic reprogramming significantly influences the progression of malignancies and the polarization of TAMs. The strategic alteration of metabolic processes has emerged as a pivotal component in the clinical management of tumors. Tumor metabolic reprogramming is a biological process in which tumor cells systematically adjust and transform their metabolic patterns to adapt to changes in the external environment and to meet the needs of their own growth and differentiation under specific physiological or pathological conditions. This process includes glucose metabolic reprogramming, lipid metabolic reprogramming, and AA metabolic reprogramming^10^. As an important component of tumor metabolic reprogramming, AA metabolic reprogramming plays an irreplaceable role in a variety of malignancies and their associated macrophage polarization processes^11^-^14^. However, TAMs and tumor cells are competitive for AA metabolic reprogramming. On the one hand, tumor cells can maintain their nutritional requirements for rapid proliferation by adjusting the expression of key enzymes of the AA metabolic pathway and AA transporter receptors; on the other hand, the uptake of AAs by tumor cells disrupts AA metabolic homeostasis in the TME, which in turn affects the utilization of AAs by TAMs and induces M2-like polarization of TAMs.

The bile duct and pancreas, as core digestive and metabolic organs in the human body, play an irreplaceable role in material metabolism. Their inherent physiological functions determine unique metabolic properties, which directly influence the metabolic regulation of tumor cells and consequently shape their metabolic preferences. Specifically, as the primary digestive gland, pancreatic acinar cells continuously synthesize and secrete multiple pancreatic enzymes to break down nutrients. Glutamine (Gln), serving as a key substrate for protein synthesis while also providing energy to pancreatic cells, is indispensable for normal pancreatic enzyme production. This creates a high physiological demand for Gln in pancreatic cells. Upon malignant transformation, tumor cells intensify Gln uptake and utilization to meet the voracious energy and material demands of rapid proliferation. This is achieved through mechanisms such as upregulating Gln transporters and related metabolic enzymes, ultimately resulting in PC cells exhibiting significantly higher Gln dependency compared to tumor cells originating from non-metabolic core organs like the lung or breast. As the bile ducts serve as crucial pathways for bile excretion, their epithelial cells participate in bile acid (BA) metabolism and transport. This specialized physiological function also shapes unique metabolic preferences in BTC cells. For instance, BTC cells exhibit heightened dependence on BA-related metabolic pathways. The distinct metabolic properties of the biliary-pancreatic organs significantly shape the metabolic phenotype of tumor cells. Concurrently, the TME of biliary and pancreatic malignant tumors exhibits distinct characteristics compared to other cancers. In biliary and pancreatic malignant tumors, TAMs constitute the most abundant and functionally pivotal immunosuppressive cells. TAMs not only directly secrete multiple cytokines to suppress effector T cells and natural killer (NK) cells activation, thereby weakening the body's antitumor immune response, but also massively produce collagenases, matrix metallopeptidase (MMP) and other substances that promote collagen deposition and cross-linking within the extracellular matrix, accelerating tumor fibrosis. The dense fibrous tissue in biliary and pancreatic malignant tumors not only diminishes therapeutic drug efficacy but also restricts the migration and infiltration of immune cells into tumor lesions. This further exacerbates immunosuppression, creating a vicious cycle of “therapeutic resistance-immunosuppression.” This cycle is a key factor contributing to the challenging treatment and poor prognosis of biliary and pancreatic malignant tumors. Therefore, in-depth investigation of the AA metabolic reprogramming in biliary and pancreatic malignant tumors and its relationship with TAMs is of great significance. In this review, we summarize how AA metabolic reprogramming contributes to the development and progression of biliary and pancreatic malignant tumors and explore the impact of AA metabolic reprogramming on the polarization of TAMs to explore new targets for metabolic cancer therapy.

## 2. Polarization Characteristics of TAMs and their Relationship to Biliary and Pancreatic Malignant Tumors

Macrophages are the major cells in the mononuclear phagocyte system. When the homeostasis of the body's internal environment is disrupted, macrophages are able to remove exogenous microorganisms by phagocytosis and release inflammatory factors to restore the dynamic balance of the body. This process is known as macrophage activation. The biological process by which macrophages change their phenotype in response to cytokines to increase their ability to cope with changes in the microenvironment is known as macrophage polarization. Macrophage polarization is important for defense against pathogens, the regulation of inflammation, the repair of tissues, and the maintenance of stable homeostasis in the body. Depending on the site of settlement, macrophages can be categorized into various types, such as microglia, hepatic Kupffer cells, alveolar macrophages and osteoclasts. Among them, macrophages recruited to the tumor region are usually referred to as TAMs. TAMs, which exhibit a high degree of plasticity and are the most abundant immune cells in the TME, are comprised of tissue-resident macrophages and bone marrow-derived macrophages that are recruited to the tumor region. TAMs can be polarized into a state between M1-like TAMs and M2-like TAMs under the induction of various signals. Among them, interferon-γ (IFN-γ), tumor necrosis factor-α (TNF-α), bacterial lipopolysaccharides (LPS) and granulocyte‒macrophage colony-stimulating factor can induce the production of M1-like TAMs. M1-like TAMs are known for their ability to promote inflammation through the release of many proinflammatory factors, such as inducible nitric oxide synthase (iNOS), reactive oxygen species (ROS), interleukin-12 (IL-12) and IL-23, which promote the T helper 1 (Th1) cell immune response and have strong proinflammatory and tumor suppressive abilities. In contrast, M2-like TAMs mainly promote tumor growth, metastasis and angiogenesis by inducing immunosuppression. In the hypoxic TME, M2-like TAMs not only drive immunosuppressive TME formation by recruiting forkhead box P3+ regulatory T cell (Treg) but also stimulate tumor angiogenesis and promote tumor progression toward malignancy by secreting Th2 cell cytokines such as IL-4, IL-13, transforming growth factor-β (TGF-β) and epidermal growth factor^15^,^16^.

Currently, several studies have shown that the development and progression of biliary and pancreatic malignant tumors are closely related to TAMs. Compared with M1-like TAMs, M2-like TAMs are dominant in cancer, and their expression levels are negatively correlated with the survival of patients^17^-^19^. Growth arrest-specific gene 6 (Gas6) is encoded by growth arrest-specific genes and is involved in biological processes such as apoptosis, differentiation, metastasis and angiogenesis of tumors through specific signaling pathways. In pancreatic ductal adenocarcinoma (PDAC), Gas6 has been reported to be produced primarily by TAMs and CAFs. Gas6 binds not only to the receptor tyrosine kinase AXL in PDAC cells, activating the Gas6/AXL signaling pathway and promoting epithelial‒mesenchymal transition (EMT), but also to the receptor tyrosine kinase AXL in NK cells, inhibiting immune cell activation and accelerating tumor cell metastasis^20^. Moreover, PC-derived exosomal FGD5 antisense RNA 1 can accelerate tumor progression toward malignancy by activating the signal transducer and activator of transcription 3 (STAT3)/nuclear factor kappa-B (NF-κB) pathway to stimulate the polarization of M2-like TAMs and produce associated inflammatory factors^21^. In addition, human cartilage glycoprotein-39 (HC-gp39) derived from M2-like TAMs can induce the expression of growth differentiation factor 15, which is secreted by tumor cells and promotes the expression of phosphatidylinositol 3-kinase (PI3K), protein kinase B (AKT) and extracellular regulated protein kinase (ERK) activation, which in turn upregulates programmed cell death protein 1 and its ligand, leading to tumor immune escape^22^. The expression level of HC-gp39 was positively correlated with tumor size and lymph node metastasis in patients with GBC. Moreover, in CCA, cysteine (Cys)-rich acidic secreted proteins can induce M2-like TAM polarization through the PI3K/AKT signaling pathway and increase the proliferation and migration of tumor cells^23^. Currently, approximately 20% of CCA patients are characterized by isocitrate dehydrogenase 1 (IDH1) mutations. Compared with that in the control group, the expression of chemokine ligand 2 (CCL2) in IDH1-mutant CCA cells has been shown to be greater. The increase in the expression level of CCL2, a class of chemokines involved in the recruitment and polarization of M2-like TAMs, also indicates that the infiltration of M2-like TAMs in IDH1-mutant CCA is increased and that the degree of tumor malignancy is aggravated^24^. More importantly, Yang L *et al.*^25^ found that the apoptosis of CCA cells was significantly reduced when lenvatinib-induced CCA cells were cocultured with M2-like TAMs. However, the apoptotic capacity of CCA cells cocultured with M1-like TAMs was greater than that of the former. These authors proposed that M2-like TAMs may inhibit the antitumor effects of lenvatinib on CCA by secreting various proangiogenic factors and modulating tumor immunity. In summary, maintaining the polarization balance of TAMs plays a crucial role in the development and progression of biliary and pancreatic malignant tumors. A comprehensive investigation into the mechanisms by which TAMs facilitate the progression of biliopancreatic malignancies is imperative for the diagnosis and treatment of patients with tumors.

## 3. AA Metabolic Reprograming in Biliary and Pancreatic Malignancies

**Figure 1:**
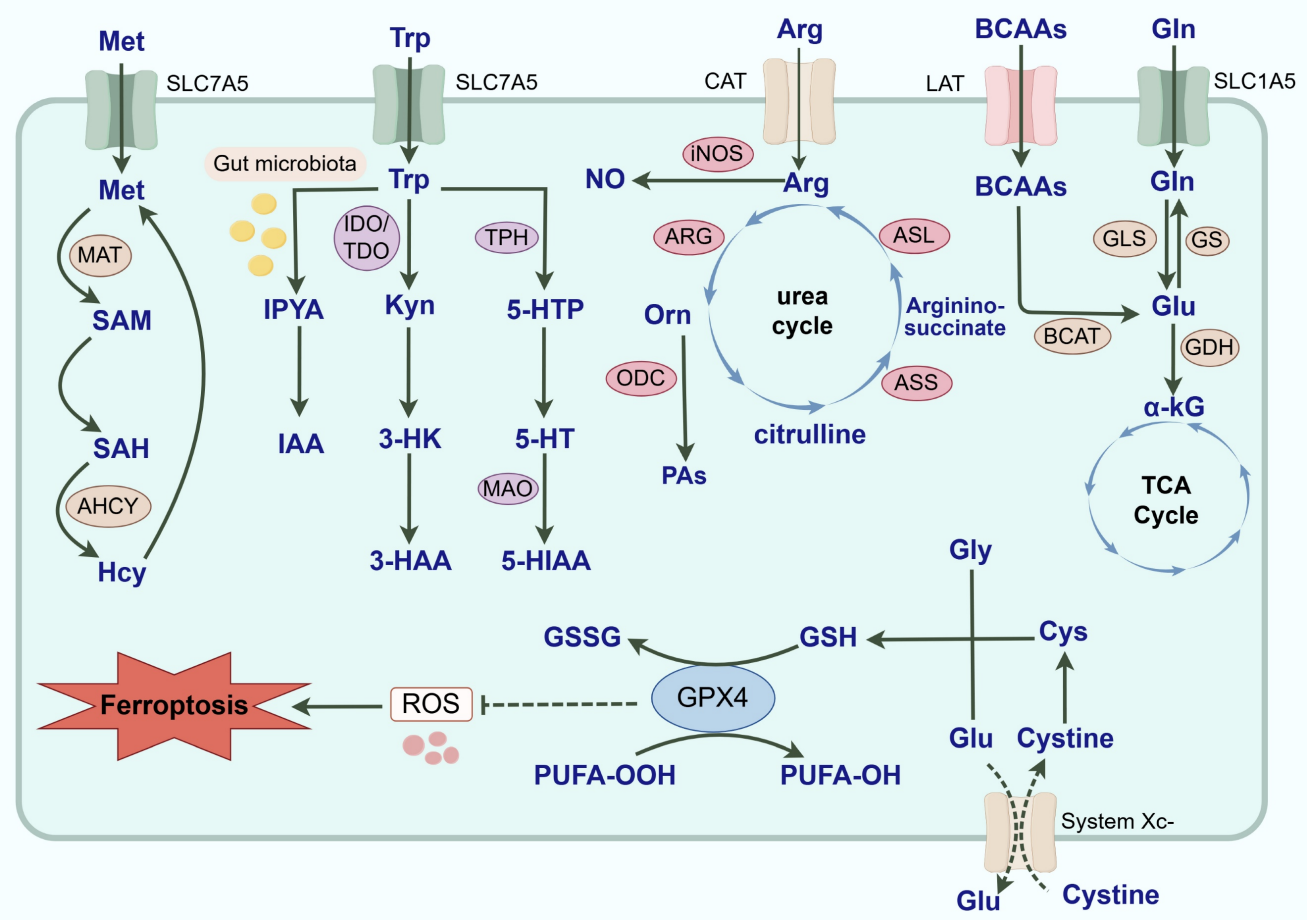
** Common AA metabolic reprogramming in tumor cells.** AA metabolic reprogramming is widely involved in the malignant progression of tumors. With the assistance of SLC1A5, Gln is transported intracellularly and catalyzed by GLS to Glu. Then, Glu can not only generate α-KG under the catalysis of GDH and participate in the TCA cycle, but also regenerate Gln under the catalysis of GS. And BCAAs mediated by LAT also generates Glu in the catalysis of BCAT. Arg is transported to cells mediated by CAT. iNOS and ARG are key enzymes in the catabolism of Arg and exert a double-edged effect on the driving of TAMs phenotype and the malignant progression of tumors by competing for the substrate Arg and generating multiple metabolites. Arg generates Orn under the influence of ARG. Orn can not only participate in the urea cycle to generate citrulline, and reproduce the synthesis of Arg under the action of ASS and ASL, but also be catalyzed by ODC to PAs. For Trp metabolic reprogramming, it is involved in the initiation and progression of tumors through the Kyn, 5-HT and indole metabolic pathways. Most Trp is catabolized through the Kyn metabolic pathway. Kyn is formed by TDO and IDO-catalyzed Trp, which is not only an endogenous agonist of AhR, but also further produces 3-HAA and 3-HK to exert biological effects. And 5-HT produced by the breakdown of Trp transduces cellular signals by binding to 5-HTR and is catabolically metabolized by MAO. Exogenously ingested Trp is typically converted to IAA and enters the TME through the bloodstream under the catalysis of the gut microbiota. Met is catalyzed by MAT to produce SAM, and SAM is converted to SAH after the methyl group is delivered to the receptor substrate. SAH can be hydrolyzed to Hcy under the catalysis of adenosine homocysteinase. And Hcy is re-methylated and generates Met through a variety of pathways. Finally, ferroptosis is regulated by iron metabolism, lipid metabolism and the system Xc^-^/GPX4 pathway. System Xc^-^ regulates GSH synthesis mainly by mediating cystine uptake and Glu output. GPX4 is able to utilize GSH to reduce PUFA-OOH to nontoxic PUFA-OH, thereby protecting cells from ferroptosis. In summary, AA metabolism reprogramming is complex and diverse, and it actively participates in the occurrence and development of tumors. (Gln: glutamine; GLS: glutaminase; GS: glutamine synthetase; SLC: solute carrier; Glu: glutamate; α-KG: α-ketoglutarate; TCA: tricarboxylic acid; GDH: glutamate dehydrogenase; BCAAs: branched-chain amino acids; BCKAs: branched-chain keto acids; BCAT: branched-chain amino acid aminotransferase; LAT: L-type amino acid transporter; Arg: Arginine; ASS: argininosuccinate synthase; ASL: argininosuccinate lyase; CAT: cationic amino acid transporter; ARG: arginase; iNOS: inducible nitric oxide synthase; NO: nitric oxide; Orn: ornithine; PAs: polyamines; ODC: ornithine decarboxylase; Met: Methionine; MAT: methionine adenosyltransferase; SAM: S-adenosylmethionine; SAH: S-adenosylhomocysteine; Hcy: homocysteine; AHCY: adenosylhomocysteinase; Trp: tryptophan; Kyn: kynurenine; 5-HT: 5-hydroxytryptamine; TDO: tryptophan 2,3-dioxygenase; IDO: indoleamine 2,3-dioxygenase; AhR: aryl hydrocarbon receptor; 3-HAA: 3-hydroxyanthranilic acid; 3-HK: 3-hydroxykynurenine; TPH: ryptophan hydroxylase; 5-HTR: 5-hydroxytryptamine receptor; s; 5-HTP: 5-hydroxytryptophan; MAO: monoamine oxidase; IAA: indole-3-acetic acid; IPYA: indole-3-pyruvic acid; 5-HIAA: 5-hydroxyindole acetic acid; GSH: glutathione; GPX: glutathione peroxidase; Cys: cysteine; Gly: glycine; GSSG: glutathione disulfide; PUFA: polyunsaturated fatty acid; AA: amino acid; TME: tumor microenvironment; TAMs: tumor-associated macrophages; ROS: reactive oxygen species).

AAs, organic compounds containing amino and carboxyl groups, are the basic constituent units of biologically functional macromolecular proteins and have vital biological functions, such as regulating redox balance, synthesizing energy and maintaining homeostasis. Depending on their source, AAs can be categorized into two main groups: essential amino acids (EAAs) and nonessential amino acids (NEAAs). While the definition of EAAs and NEAAs is appropriate for normal cells, the classification does not apply to cancer cells^26^. Altered AA metabolism is common in tumors, and NEAAs usually become indispensable in tumors. At present, an increasing number of studies have shown that AA metabolic reprogramming is inseparable from the progression of tumors to malignancy^27^. For example, Bodineau C *et al.*^28^ found that Gln-dependent tumors significantly increased glutaminase (GLS) activity through activation of the mammalian target of rapamycin (mTOR) signaling pathway to meet the energy requirements of organisms. Moreover, melanoma and breast cancer (BC) gain a growth advantage via the upregulation of enzymes associated with the serine (Ser) synthesis pathway^29^. Therefore, an in-depth investigation of the biological effects of AA metabolic reprogramming on biliary and pancreatic malignant tumors is important (Fig. 1).

### 3.1. Glutamine

Gln is an important nitrogen donor and carbon donor with a variety of biological functions 30. Gln can be ingested by food or synthesized by glutamine synthetase (GS). Gln that enters the organism is transported into the cell with the assistance of members of the solute carrier (SLC) family and is catabolized in the mitochondria to produce glutamate (Glu), which is catalyzed by GLS. Glu is then converted to α-ketoglutarate (α-KG) and produces acetyl-coenzyme A to promote the tricarboxylic acid (TCA) cycle, which participates in the energy metabolism of tumors. Gln is the fastest consumed AA in tumor cells. Tumor cells utilize Gln avidly, known as Gln addiction^31^. In addition, Glu can also be used to synthesize glutathione (GSH) to maintain redox homeostasis in tumor cells. When GLS-1 expression is downregulated, the GSH content decreases, the ROS level increases, and the cells exhibit redox imbalance. Downregulation of GLS-1 expression promotes the degradation of glutathione peroxidase 4 (GPX4), which ultimately induces ferroptosis^32^. Moreover, Gln can be metabolized into intermediates such as carbamoyl phosphate and phosphoribosyl amine for the synthesis of purines and pyrimidines, which are essential components for deoxyribonucleic acid (DNA) synthesis and repair during rapidly tumor proliferation^33^. At present, an increasing number of reports have indicated that the progression of biliary and pancreatic tumors to malignancy is closely related to Gln metabolic reprogramming. For example, patients with intrahepatic cholangiocarcinoma (ICC) had more Gln in their metabolites than healthy patients did. The chemoresistance of these patients was positively correlated with SLC1A5 and GLS-1 expression levels^34^. And GLS is highly expressed in PDAC. Succinylation of GLS has been demonstrated to increase Gln catabolism and drive the malignant proliferation of PDAC cells^35^.

Myelocytomatosis (Myc) acts as a vital transcriptional regulator, and its aberrant activation could affect the nutrient uptake and drug resistance of tumor cells by regulating key metabolic enzymes or metabolic pathways^36^,^37^. Gln metabolism has extremely strong effects for the cellular myelocytomatosis (c-Myc)-induced mouse liver tumor cells^38^. In PC cells with high expression of mucin 5AC (MUC5AC), disruption of E-cadherin/β-catenin junctions and subsequent promotion of β-catenin nuclear translocation were observed. This resulted in the upregulation of c-Myc and the increase in Gln uptake, which ultimately drove tumor progression and resistance to gemcitabine (GEM)^39^. Wappler J *et al.*^40^ demonstrated that platinum resistance is induced by high c-Myc levels through increased utilization of Gln in CCA. At the same time, Gln metabolism reprogramming actively participates in ferroptosis of PDAC and CCA. It was found that when activin receptor-like kinase 5 (ALK5) and nicotinamide adenine dinucleotide phosphate oxidase 1 (NOX1) are overexpressed, the expression of ROS and Fe2^+^ is upregulated, and the expression of SLC7A11 and GSH is downregulated, which in turn induces ferroptosis. However, Gln supplementation could reverse this effect by downregulating ALK5 and NOX1^41^. Targeting Gln dependency endows PC cells with susceptibility to GPX4-dependent ferroptosis through epigenetic remodeling^42^. However, there are certain differences in the dependence mechanism of BTC and PC on Gln metabolic reprogramming. More than 90% of PDAC patients carry activating mutations in the oncogene Kirsten rat sarcoma viral oncogene homolog (KRAS), a major driver of Gln addiction. It was found that PDAC in mutant KRAS is dependent on a unique Gln metabolic pathway in which Gln-derived aspartate is transported into the cytoplasm where it can be converted into oxaloacetate by aspartate transaminase. Subsequently, this oxaloacetate is converted into malate and then pyruvate, ostensibly increasing the nicotinamide adenine dinucleotide phosphate hydrogen (NADPH)/nicotinamide adenine dinucleotide phosphate (NADP+) ratio which can potentially maintain the cellular redox state^43^. In contrast, BTC is more heterogeneous in terms of driver mutations for Gln metabolism reprogramming. Approximately 20% of CCA patients have IDH1/2 mutations. The carcinogenic metabolite D-2-hydroxyglutarate (D-2-HG) produced by IDH mutations induces DNA and histone hypermethylation by competitively inhibiting the activity of α-KG-dependent epigenetic regulatory enzymes, thereby driving tumorigenesis and development, becoming the core mechanism of CCA malignant transformation^44^. In addition, a distinctive feature of PDAC compared to CCA is that it has a dense fibrotic interstitium. Pancreatic stellate cells (PSCs) are the main effector cells of PC fibrosis. PSCs participate in the regulation of TME and the signal transmission between PC cells, which is crucial for the metabolism and progression of PDAC. Liu H *et al.*^45^ found that PSCs promote PC cell growth through Wnt/β-catenin/TCF7-mediated Gln metabolism. At the same time, Yes-associated protein (YAP) regulates the expression of GLS-1 in PSCs by upregulating Myc. The Yap/Myc signaling pathway may be a therapeutic target for connective tissue hyperplasia in PDAC^46^. Moreover, for BTC, its unique ductal structure allows BTC cells to come into direct contact with bile. BAs, as endogenous pro-inflammatory and pro-oxidative molecules, can drive oxidative stress by upregulating ROS^47^. Lithobionic acid, a secondary BA produced by the metabolism of the intestinal flora, is a tumor suppressor in GBC. LCA inhibits key enzymes involved in Gln metabolism and induces oxidative stress, triggering ferroptosis^48^. Therefore, BTC cells exposed to BAs may upregulate Gln uptake and metabolism to generate GSH to counteract oxidative stress and protect themselves from injury. This TME-driven alteration of metabolic adaptation is a key feature that distinguishes BTC from PDAC and suggests that metabolic therapeutic strategies targeting BTC may focus more on antioxidant pathways. In summary, Gln metabolic reprogramming plays a central role in the pathogenesis, progression, and drug resistance of biliary and pancreatic malignancies.

### 3.2. Tryptophan

Tryptophan (Trp) can participate in the development and progression of biliary and pancreatic malignant tumors through the kynurenine (Kyn), 5-hydroxytryptamine (5-HT) and indole metabolic pathways. Most dietary Trp is catabolized through the Kyn metabolic pathway. Kyn is formed by Trp catalyzed by tryptophan 2,3-dioxygenase (TDO) and indoleamine 2,3-dioxygenase (IDO) and is not only an endogenous agonist of the aryl hydrocarbon receptor (AhR) but also produces 3-hydroxyanthranilic acid (3-HAA) and 3-hydroxykynurenine (3-HK) to exert biological effects. Compared with those in normal pancreatic tissues, IDO expression levels are significantly elevated in PC and are correlated with poor patient prognosis^49^. Wang L *et al.*^50^ reported that the level of Kyn was associated with high iNOS expression and that a higher level of Kyn predicted poor survival in PDAC patients. They reported that nitric oxide (NO) upregulated runt-related transcription factor 3 (RUNX3), which could increase the ability of IDO1 to catalyze the catabolism of Trp and produce Kyn. A persistent influx of Kyn into the AhR has the potential to promote malignant progression and chemoresistance in PDAC. Moreover, the serum levels of 3-HAA and the 3-HAA/3-HK ratio are negatively correlated with the risk of developing PC^51^. In GBC, it was found that IFN-γ induces the expression of IDO through the Janus kinase (JAK)/STAT1 signaling pathway, thereby inhibiting the activation and proliferation of T cells, resulting in immune tolerance of the local microenvironment. Suberoylanilide hydroxamic acid (SAHA) can down-regulate the expression of IDO via inhibition of the JAK/STAT1 signaling pathway in GBC^52^. However, compared to the malignant progression of PDAC that relies on the Trp/Kyn pathway, BTC is more dependent on the Trp/5-HT pathway. Trp can generate 5-HT via a process catalyzed by tryptophan hydroxylase (TPH) and 5-hydroxytryptophan decarboxylase^53^. 5-HT transduces cellular signals by binding to the 5-hydroxytryptamine receptor (5-HTR) and is catabolically metabolized by monoamine oxidase (MAO). 5-HT concentrations could be a potential diagnostic indicator of CCA^54^. Compared with those in normal bile duct epithelial cells, the expression levels of TPH1,5-HT1A, 5-HT2A/AB and 5-HT4/6 are elevated, and the expression level of MAO-A is decreased in CCA cells^55^. When the promoter region of MAO-A is highly methylated, the catabolic capacity of 5-HT *in vivo* decreases, and the growth of CCA cells slows^56^. In contrast, in PDAC, the pro-tumorigenic effect of 5-HT is more focused on directly reprogramming the metabolism of tumor cells to drive proliferation through HTR2B receptor signaling, upregulation of glycolysis, and the pentose phosphate pathway^57^. Moreover, Tintelnot J *et al.*^58^ found that the microbiota-derived Trp metabolite indole-3-acetic acid (IAA) was enriched in PDAC patients who responded to chemotherapy. When catalyzed by intestinal microorganisms, such as *Bacteroides fragilis* and *Bacteroides multiforme*, exogenously ingested Trp was often converted to IAA and entered the TME via the bloodstream. IAA synergistically induced the downregulation of GPX3 and GPX7 in conjunction with chemotherapeutic agents to promote the accumulation of ROS, which then inhibited autophagy and increased the efficacy of chemotherapy in PDAC. This study identified a group of clinically meaningful microbiota-derived metabolites for PDAC therapy. In summary, although the Trp metabolic reprogramming in both BTC and PC involves the Kyn, 5-HT and indole metabolic pathways, different types of tumors exhibit distinctly different enzyme expression profiles, signaling pathways and downstream biological effects.

### 3.3. Arginine

Arginine (Arg) is a conditionally NEAA, and its homeostasis relies on the dynamic balance of exogenous intake, endogenous synthesis and catabolism. In the synthetic pathway, the key enzymes of the urea cycle - argininosuccinate synthetase-1 (ASS1) and argininosuccinate lyase (ASL) - co-catalyze the conversion of citrulline to Arg. Currently, most studies have shown that tumor cells have a high demand for Arg. ASS1, a rate-limiting enzyme for Arg synthesis, is generally highly expressed in normal tissues and a few tumors, such as those of colorectal cancer (CRC). However, biliary and pancreatic malignant tumors tend to reduce endogenous synthesis of Arg by downregulating ASS1, making them dependent on exogenous uptake of Arg to satisfy their energy requirements for rapid growth^59^. This particular neoplasm is also referred to as the Arg nutrition-deficient tumor. It has been shown that the defective expression of ASS1 is closely related to the hypermethylation status of its promoter region^60^. In response to Arg depletion in TME, p53 can increase exogenous Arg uptake by upregulating SLC7A3, a mechanism that drives the malignant progression of PDAC^61^. In catabolism, arginase (ARG) hydrolyzes Arg to ornithine (Orn) and urea. Orn can further generate putrescine, spermidine and spermine catalyzed by ornithine decarboxylase (ODC). Arg catabolism is widely involved in the malignant progression of tumors. For example, ARG1 drives tumor immune suppression by consuming Arg and inhibiting T cell activation^62^. In ICC, ARG1 has emerged as an independent prognostic biomarker, and its expression is positively correlated with poor patient prognosis^63^. And the ARG2 level is closely related to the body mass index. Obesity, as an important risk factor for PC, can lead to a large accumulation of ammonia by upregulating ARG2, thereby promoting the malignant progression of PDAC^64^. Moreover, CAFs highly express ARG2 in highly fibrotic PDAC. Ino Y *et al.*^65^ found that these CAFs expressing ARG2 interact with hypoxia inducible factor-1α (HIF-1α) in PDAC and are closely associated with shorter overall survival in PDAC patients. In addition, gallbladder mucosa ODC activity is increased in patients with anomalous arrangement of the pancreaticobiliary duct, which may contribute to the pathogenesis of GBC^66^. Notably, polyamine metabolism exhibits unique adaptations in PDAC. PDAC often increases polyamine production and transport to maintain high growth rates^67^. However, Lee MS *et al.*^68^ found that, unlike other tumor cells that primarily utilize Arg to synthesize Orn and polyamines (PAs), PDAC cells are more reliant on Gln as a raw material for synthesizing Orn and PAs. This dependency is related to the depletion of Arg in PDAC and is driven by mutated KRAS. On one hand, the myeloid cells expressing ARG1 deplete Arg in the TME through large-scale degradation; on the other hand, mutated activated KRAS can induce the expression of ornithine aminotransferase (OAT) and polyamine synthase, thereby promoting the synthesis of Gln into Orn. Furthermore, methylation modification of Arg residues is an important regulatory mechanism for the occurrence and development of CCA and PDAC. Elurbide J *et al.*^69^ reported that arginine methyltransferase 5 (PRMT5) and methylated epitope protein 50 (MEP50) were highly expressed in CCA, which was closely related to the methylation of Arg, and that targeting PRMT5 had significant antitumor efficacy in clinically relevant CCA models. And PRMT1 promotes epigenetic reprogramming associated with acquired chemoresistance in PC^70^. In summary, Arg metabolic reprogramming plays an irreplaceable role in the genesis and progression of biliopancreatic malignant tumors through the three levels of synthetic inhibition, catabolic activation and modification regulation.

### 3.4. Methionine

Methionine (Met) is the only sulfur-containing EAA in living organisms. Met is catalyzed by methionine adenosyltransferase (MAT) to produce S-adenosylmethionine (SAM), which participates in the methylation of many active substances. SAM is converted to S-adenosylhomocysteine (SAH) after the methyl group is delivered to the receptor substrate. SAH can be hydrolyzed to homocysteine (Hcy) via the catalysis of adenosine homocysteinase. Hcy is then remethylated and generates Met through a variety of pathways. The above metabolic process is known as the Met cycle. Met metabolism reprogramming has been shown to be different between normal cells and tumor cells. Many studies have demonstrated that the restriction of Met intake has the potential to effectively inhibit tumor growth and increase the sensitivity of tumors to treatment. Moreover, the tumorigenic capacity is significantly reduced when Met is removed or replaced by its immediate precursor Cys^71^,^72^. The absolute need for Met by tumor cells is known as Met addiction or the Hoffman effect. Although most studies suggest that Met metabolic reprogramming plays an integral and important role in the malignant progression of BTC and PC, BTC and PC show significant differences in Met metabolic dependence and regulatory mechanisms associated with their respective TME. Met metabolic reprogramming in PDAC shows features closely related to its highly fibrotic and hypoxic nature. The malignant growth of PDAC is associated with complex redox signaling mechanisms. As an AA susceptible to oxidative modification, the Met residues are regulated by methionine sulfoxide reductase A (MSRA). Met residues can act as reversible redox switches to control different signaling pathways. Deletion of MSRA led to the selective oxidation of the Met residue M239 in pyruvate kinase M2 (PKM2) and thus accelerated the metastasis of PDAC cells^73^. This highlights the specificity of redox regulation in the reprogramming of Met metabolism in PDAC. At the same time, PC cells with mutant KRAS require strong basal autophagy for viability and growth. Montenegro MF *et al.*^74^ observed that some processes that allow the maintenance of basal autophagy in pancreatic cancer cells are controlled by protein methylation. Hypomethylation treatment can disrupt protein methylation and induces SAH accumulation while depleting cellular SAM levels, leading to inhibition of autophagy and endoplasmic reticulum stress-induced apoptosis in PC cells. p53 is a commonly mutated gene in PC. Panatta E *et al.*^75^ suggested that inactivation of p53 reduced the synthesis of SAM in tumor cells by regulating the transcription of SLC43A2. In addition, activated PSCs are highly dependent on methyl provided by SAM to synthesize large amounts of extracellular matrix. In contrast, Met metabolic reprogramming in BTC is more significantly regulated by epigenetic mechanisms. MAT1A is regarded as an oncogene, and its expression is diminished in CCA^76^. Avila MA *et al.*^77^ observed the hypermethylation of the MAT1A promoter DNA in CCA, which plays an important role in MAT1A silencing. In addition, prohibitin 1 (PHB1) is an evolutionarily highly conserved protein. To date, researchers have reported that PHB1 is aberrantly expressed in a variety of malignant tumors, including glioblastoma, CRC and prostate cancer^78^-^80^. PHB1 is strongly correlated with mitochondrial dysfunction, oxidative stress injury, cell proliferation and apoptosis. Mice lacking MATα1 tend to have lower PHB1 expression. Fan W *et al.*^81^ found that PHB1 was highly expressed in normal hepatocytes and bile duct epithelial cells but downregulated in hepatocellular carcinoma (HCC) and CCA. When PHB1 expression was downregulated, the expression level of MAT1A decreased, and the expression levels of c-Myc, MAF bZIP transcription factor G (MAFG) and cellular musculoaponeurotic fibrosarcoma (c-MAF) increased; these proteins then bound to the E-box promoter region of the MAT1A blocker, negatively regulating the transcription of MAT1A and ultimately promoting the proliferation of tumor cells. Moreover, SAM can block GBC cell proliferation and induce apoptosis by inhibiting the JAK2/STAT3 signaling pathway^82^. In conclusion, Met metabolic reprogramming is highly important for biliary and pancreatic malignancies.

### 3.5. Branched-chain amino acids

Branched-chain amino acids (BCAAs), including valine, leucine (Leu) and isoleucine, have important biological functions, as they are not only directly involved in protein synthesis but also can be decomposed into branched-chain keto acids (BCKAs) and Glu, which are catalyzed by branched-chain amino acid aminotransferase (BCAT) to participate in the TCA cycle and nucleotide synthesis. BCAA metabolic reprogramming plays a key role in CCA and PC, but there are differences in metabolic regulatory mechanisms and TME interactions between the two. A number of studies have demonstrated that the expression of BCAAs, SLC7A5 and its accessory protein cluster of differentiation 98 (CD98) is upregulated in patients with CCA and correlated with poor prognosis^83^-^85^. When SLC7A5 was knocked down in CCA cells, microRNA-7 expression was upregulated, the 4F2hc signaling pathway was inhibited, and tumor progression to malignancy was delayed^86^. Currently, Leu is considered an EAA for the activation of mTORC1, and its metabolic reprogramming is finely regulated in CCA. Li Z *et al.*^87^ reported that pancreatic progenitor cell differentiation and proliferation factor (PPDPF) was upregulated in CCA. It could block the interaction between 3-methylcrotonyl-CoA carboxylase A (MCCA) and MCCB, inhibit Leu catabolism and activate mTORC1 signaling, thereby increasing the malignant phenotype of CCA cells. And the highly activated IL-6/STAT3 signaling pathway in BTC may exacerbate metabolic reprogramming of tumors by transcriptionally upregulating SLC7A5^88^. These findings suggest that the BCAA metabolic reprogramming in CCA focuses more on the uptake and signaling of BCAAs. In stark contrast, the BCAA metabolic reprogramming in PDAC is more complex. On one hand, similar to CCA cells, PDAC cells also exhibit abnormal activation of BCAA metabolism. A diet with a high content of BCAAs can promote the malignant progression of PDAC by stabilizing ubiquitin specific peptidase 1 (USP1)-mediated BCAT2^89^. At the same time, BCAA metabolic reprogramming is profoundly intertwined with KRAS in PDAC. It has been show that in KRAS-mutant PDAC, spleen tyrosine kinase (Syk) and tripartite motif containing 21 (TRIM21) accelerate tumor progression toward malignancy by regulating the ubiquitination of BCAT2 to maintain the catabolism and mitochondrial respiration of BCAAs^90^. On the other hand, compared to CCA, the content of CAFs in PC cells is higher. Stromal cells play a major role in alleviating the nutritional deficiencies of PDAC cells. Zhu Z *et al.*^91^ found that the catabolic flux of BCAAs was significantly increased in CAFs, and their secreted BCKAs could be used to enhance protein synthesis, TCA cycling and oxidative phosphorylation in PC cells. And in PDAC, the activation of mTORC1 may be more directly associated with the overall anabolic metabolism driven by KRAS and the nutritional support provided by CAFs. In summary, there is a significant difference between BTC and PC in the utilization of BCAAs: CCA primarily manifests as the autonomous uptake of BCAAs and activation of signaling pathways by tumor cells, whereas PDAC has evolved a dual mechanism that includes both the catabolism of BCAAs by tumor cells and nutritional support from stromal cells. This fundamental difference profoundly reflects the disparity between the two types of tumors in terms of TME and metabolic adaptability, and provides a theoretical basis for the more precise development of treatment methods.

### 3.6. Other AAs

In addition to the AAs described above, many other AAs, such as Cys, Ser and glycine (Gly), are actively involved in the development and progression of biliary and pancreatic malignant tumors. As a key enzyme in Cys catabolism, cysteine dioxygenase 1 is closely related to the poor prognosis of tumors and could be used as an effective indicator for the diagnosis and prognosis of CCA, GBC and PC^92^-^94^. In terms of redox regulation, biliary and pancreatic malignancies exhibit a high dependence on Cys metabolism reprogramming and are closely related to ferroptosis. Cystine and Cys expression downregulation induces cell ferroptosis by regulating GSH levels. By evaluating potential biomarkers in bile for diagnosing extrahepatic cholangiocarcinoma (ECC) and benign biliary tract disease, Han JY *et al.*^95^ found that redox-dependent modification of Cys and ferroptosis in bile might be mechanisms of ECC development. Downregulation of SLC7A11 in CCA inhibits the metabolic pathway of Cys, leading to a decrease in intracellular cystine levels and depletion of GSH biosynthesis, and indirectly inhibits GPX4 activity, which in turn leads to the accumulation of lipid peroxides, ultimately inducing ferroptosis and delaying tumor progression^96^. Similarly, Cys depletion can upregulate ferroptosis by reducing the synthesis of GSH and coenzyme A and delay the progression of PDAC toward malignancy 97. In addition, PDAC is regulated by more complex mechanisms. There is a study has shown that mitochondrial calcium uniporter can activate the Kelch-like ECH-associated protein 1 (Keap1)/nuclear factor erythroid 2-related factor 2 (Nrf2) signaling pathway and enhance tumor progression and metabolic stress resistance in a Cys-dependent manner^98^. Autophagy is a vital self-protection mechanism of cells, which helps PDAC cells to evade immunity by consuming cytotoxic T lymphocytes (CD8+ T cells) or reducing surface MHC-I expression^99^. Mukhopadhyay S *et al.*^100^ found that loss of autophagy specifically results in intracellular Cys depletion under nutrient-replete conditions. PDAC cells utilize the autophagy machinery to regulate the activity and localization of the cystine transporter SLC7A11 at the plasma membrane. Upon inhibition of autophagy, SLC7A11 is localized to lysosomes in an mTORC2-dependent manner. In addition, Ser and Gly are substrates for the synthesis of biological macromolecules, the maintenance of redox states and methylation reactions. Most tumors rely on Ser/Gly metabolic reprogramming to obtain a malignant phenotype. In the endogenous synthesis pathway of tumor cells, 3-phosphoglyceric acid, an intermediate product of glycolysis, reacts enzymatically to produce Ser, which is then catalyzed by Ser hydroxymethyltransferase to produce Gly. It has been reported that neuronal peripheral axons release Ser to maintain the growth of PDAC cells in the absence of Ser/Gly^101^. Moreover, phosphoglycerate dehydrogenase (PHGDH), a rate-limiting enzyme in the Ser/Gly metabolic pathway, is upregulated in ICC in association with histone methyltransferase G9a, suggesting that activation of the Ser/Gly metabolic pathway is involved in the progression of ICC to malignancy^102^.

In summary, AA metabolic reprogramming is actively involved in the occurrence and development of biliary and pancreatic malignant tumors (Table 1). Elucidation of the mechanisms associated with AA metabolic reprogramming is imperative.

## 4. The Mechanism of AA Metabolic Reprogramming in the Regulation of TAM Polarization

**Figure 2:**
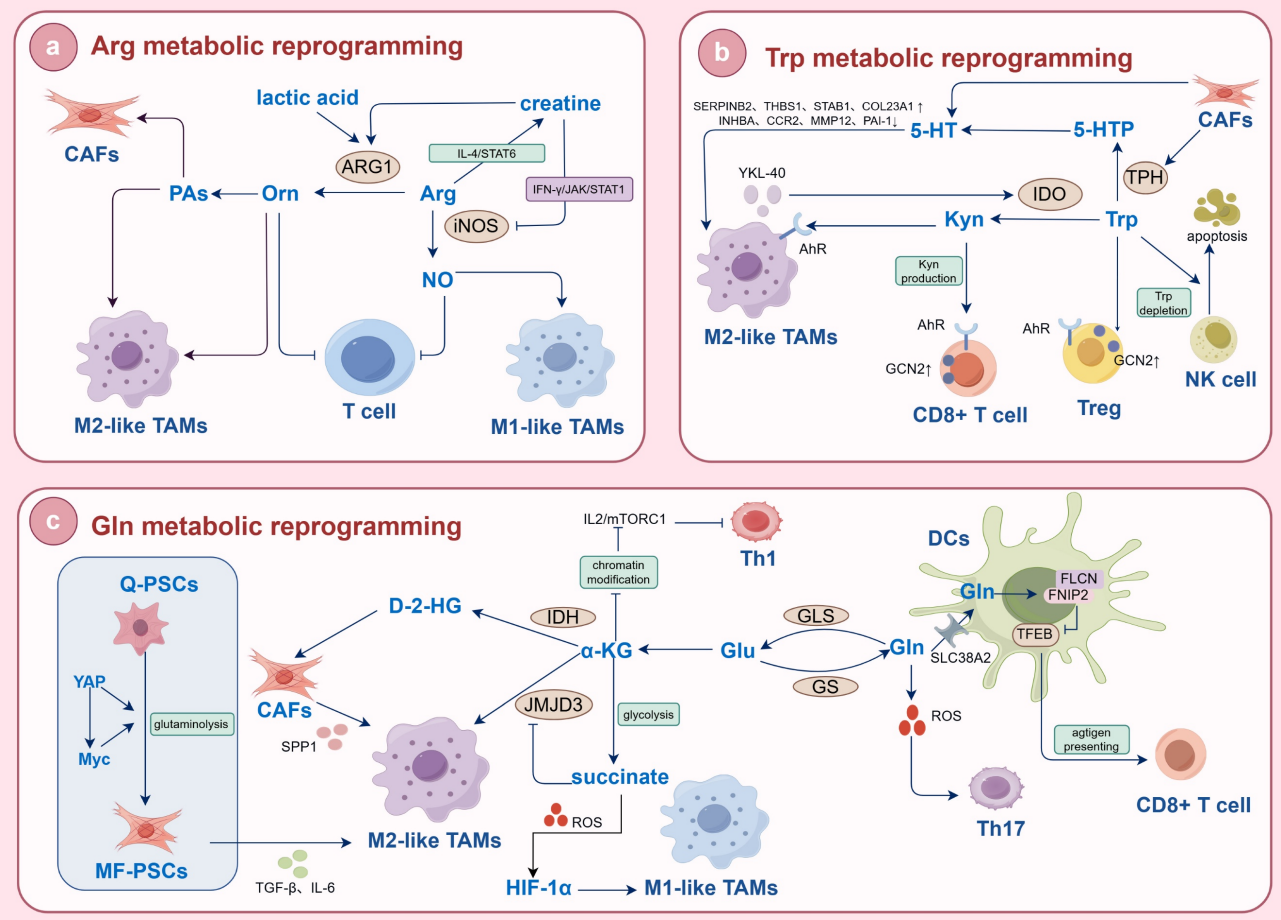
** AA metabolism reprogramming affects immune cells.** (a) Arg metabolic reprogramming. iNOS catabolizes Arg into NO, promoting the polarization of M1-like TAMs and inhibiting T cells. As the tumor progresses, the activity of ARG1 is upregulated, which catalyzes the decomposition of Arg into Orn. This process promotes the polarization of M2-like TAMs and inhibits T cells. PAs generated from Orn can not only drive the polarization of M2-like TAMs but also promote the activation of CAFs, thereby exacerbating tumor fibrosis. Meanwhile, Arg is also decomposed into creatine, which blocks iNOS activity, activates ARG, and further promotes M2-like TAM polarization. (b) Trp metabolic reprogramming. YKL-40 secreted by M2-like TAMs can upregulate IDO1 to activate the Trp/Kyn metabolic pathway. The upregulation of IDO1 leads to the depletion of Trp and accumulation of Kyn, which in turn activates GCN2 and AhR in immune cells. This activation results in the polarization of M2-like TAMs, functional impairment of CD8+ T cells, and differentiation of Treg. In addition, the depletion of Trp can also induce the apoptosis of NK cells. TPH can catalyze Trp to produce 5-HT. Meanwhile, CAFs can express TPH1 and actively secrete 5-HT, which activates 5-HTR and induces the polarization of M2-like TAMs. (c) Gln metabolic reprogramming. α-KG produced by Gln metabolism can activate JMJD3 to reduce the accumulation of H3K27me3, thereby activating M2-like TAMs. By contrast, α-KG can also participate in glycolysis to generate succinate. Succinate not only acts as an inhibitor of JMJD3, but also promotes the production of ROS and stabilizes HIF-1α, inducing the polarization of M1-like TAMs. In PC, the Yap/Myc signaling pathway can regulate Gln catabolism, induce the activation of PSCs, and drive the polarization of M2-like TAMs. In CCA, mutated IDH catalyzes α-KG to produce D-2-HG, which further induces CAFs to secrete SPP1 and thereby facilitates M2-like TAM recruitment. DCs take up Gln via SLC38A2 to promote the formation of the FLCN-FNIP2 complex. This complex restricts the activity of TFEB, thereby enhancing antigen cross-presentation and the activation of CD8+ T cells. Meanwhile, Gln metabolism exerts a unique effect: it promotes the differentiation of Th17 cells while restricting the differentiation of Th1 cells and CD8+ T cells. (iNOS: inducible nitric oxide synthase; Arg: arginine; NO: nitric oxide; TAMs: tumor-associated macrophage; ARG1: arginase; Orn: ornithine; PAs: polyamines; CAFs: cancer-associated fibroblasts; YKL-40: chitinase 3-like protein 1; IDO1: indoleamine 2,3-dioxygenase 1; Trp: tryptophan; Kyn: kynurenine; GCN2: general control nonderepressible 2 kinase; AhR: aryl hydrocarbon receptor; Treg: regulatory T Cell; NK: natural killer; TPH: tryptophan hydroxylase; 5-HT: 5-hydroxytryptamin; 5-HTR: 5-hydroxytryptamine receptor; α-KG: α-Ketoglutarate; Gln: glutamine; JMJD3: Jumonji domain-containing protein 3; H3K27me3: trimethylated histone H3 lysine 27; ROS: reactive oxygen species; HIF-1α: hypoxia inducible factor-1α; PC: pancreatic cancer; Yap: Yes-associated protein; Myc: myelocytomatosis; PSCs: pancreatic stellate cells; CCA: cholangiocarcinoma; IDH: isocitrate dehydrogenase; D-2-HG: D-2-hydroxyglutarate; SPP1: secreted phosphoprotein 1; DCs: dendritic cells; SLC: solute carrier family; FLCN: folliculin; FNIP2: folliculin interacting protein 2; TFEB: transcription factor EB; Th: T helper; IL: interleukin; STAT: signal transducer and activator of transcription; IFN-γ: interferon-γ; JAK: Janus kinase; SERPINB2: serine protease inhibitor 2; THBS1: thrombospondin 1; STAB1: stabilin 1; COL23A1: collagen type XXIII alpha 1 chain; INHBA: inhibin subunit beta A; CCR2: C-C motif chemokine receptor 2; PAI-1: plasminogen activator inhibitor-1; MMP12: matrix metallopeptidase 12; mTORC1: mechanistic target of rapamycin complex 1; CD8+ T cells: cytotoxic T lymphocytes).

Tumor cells not only induce immune tolerance by secreting extracellular signals but also compete vigorously with immune cells for the uptake of AAs and release bioactive metabolic products, collectively shaping an immunosuppressive microenvironment. Thus, in the TME, AA metabolic reprogramming has become a core mechanism linking tumor progression and immune evasion. Tumor immunity is closely related to AA metabolic reprogramming (Fig. 2). For example, GLS plays a key role in T cell activation. Gln metabolic reprogramming has distinct roles to promote Th17 cells but constrain Th1 cells and CD8+ T cells effector cell differentiation^103^. GLS deficiency not only reduces the activation and proliferation of naïve T cells and impairs the differentiation of Th17 cells, but also increases T-box expressed in T cells to promote the differentiation and effector functions of Th1 cells and CD8+ T cells. This is related to altered chromatin accessibility and gene expression, including the reduction of phosphoinositide-3-kinase interacting protein 1 in Th1 cells, making them more sensitive to IL-2-mediated mTORC1 signaling. However, DCs uptake Gln through SLC38A2 to promote the formation of the folliculin (FLCN)-folliculin interacting protein 2 (FNIP2) complex, thereby limiting TFEB (transcription factor EB) activity, facilitating antigen cross-presentation, and activating CD8+T cells^104^. In other words, AA metabolic reprogramming has a complex regulatory role in immune responses with a certain balance. For TAMs, this breakdown of AA homeostasis directly regulates the polarization and immune function. For instance, the accumulation of Glu, Kyn and taurine actively suppresses M1-like TAM polarization. A pivotal player in this process is Arg metabolism. Given that many tumors are auxotrophic for Arg, the high expression of ARG1 in TAMs creates a dual immunosuppressive mechanism: it both starves cytotoxic T cells of this essential nutrient, impairing their function, and the resulting metabolic products and nutrient stress signals directly instruct TAMs to differentiate into a pro-tumorigenic M2-like phenotype. Consequently, the expression level of Arg-metabolizing enzymes serves as a key indicator of TAMs' immunosuppressive function. In summary, AA metabolic reprogramming coordinately drives TAM polarization toward an immunosuppressive state through nutrient depletion, metabolite-mediated signaling and microenvironment acidification, thereby fostering immune evasion and tumor progression. This integrated mechanism provides a strong rationale for targeting tumor metabolic-immune crosstalk as a novel therapeutic strategy.

### 4.1. Glutamine

Gln not only provides nutrients for tumor cell survival and proliferation but also facilitates the polarization of TAMs and the induction of desirable immune responses^105^,^106^. In the TME, tumor cells compete with TAMs for the utilization of Gln. The availability of Gln affects the polarized phenotype of TAMs. When the available Gln content in the body is sufficient, TAMs undergo M2-like polarization and exert pro-cancer effects. In contrast, when the Gln content is low, TAMs undergo M1-like polarization and inhibit the progression of tumors toward malignancy 107,108. GS, a key enzyme in the synthesis of Gln from Glu, can maintain the development of the M2-like phenotype in TAMs. Knockdown of GS in a mouse model of lung cancer reversed the formation of M2-like TAMs and transformed them toward M1-like TAMs^109^. In addition, Gln metabolism generates α-KG, which activates M2-like TAMs by reducing the accumulation of trimethylated histone H3 lysine 27 (H3K27me3) through the activation of the histone demethylase Jumonji domain-containing protein 3 (JMJD3) and participates in fatty acid synthesis, assisting in the membrane reconstruction and paracrine activity of M2-like macrophages 110. And increased α-KG expression can inhibit the NF-κB signaling pathway in a prolyl hydroxylase (PHD)-dependent manner, modulate inhibitor kappa B kinase β (IKKβ) activity, and attenuate proinflammatory responses in M1-like macrophages^111^. As a proinflammatory metabolite, succinate is an inhibitor of JMJD3 and can stabilize the transcription factor HIF-1α by inhibiting PHD activity and promoting ROS production^112^.

When HIF-1α is overexpressed, the glycolytic metabolism of macrophages is increased, which induces the polarization of M1-like macrophages^113^. Thus, when the α-KG/succinate ratio is decreased, M1-like TAMs exhibit increased activation, whereas an increase in the α-KG/succinate ratio promotes M2-like TAM polarization. This regulation makes α-KG as a checkpoint for Gln catabolism. This also suggests an environment-dependent effect of Gln metabolic reprogramming on macrophage polarization. Inflammatory intensity may be a key switch that regulates the flow of Gln metabolism. Up-regulation of Gln metabolic reprogramming can rely on the α-KG/JMJD3 pathway to promote M2-like macrophage polarization, whereas inflammatory stimuli can contribute to M1-like polarization of macrophages. Available reports confirm that Gln metabolic reprogramming is actively involved in TAM polarization in biliopancreatic malignancies and that its regulatory mechanisms are closely related to tumor properties. Zhang D *et al.*^46^ found that Yap/Myc signaling induced PSCs activation by regulating Gln catabolism. Activated PSCs secrete various cytokines such as TGF-β and IL-6 to drive the polarization of M2-like TAMs^114^. In other words, the malignant progression of PC may also be a dynamic process of interaction between PSCs and TAMs under the regulation of Gln metabolism reprogramming. In CCA, TAM polarization is associated with IDH1/2 mutations. Zabransky D *et al.*^115^ showed that the IDH1-mutant CCA tumor immune microenvironment is characterized by an increase in M2-like TAMs. IDH1/2 mutation promotes the production of D-2-HG from Gln. The most upregulated gene in D-2-HG conditioned fibroblasts is secreted phosphoprotein 1 (SPP1), which has been implicated in the recruitment and polarization of immunosuppressive M2-like TAMs leading to decreased antitumor immunity. In conclusion, biliopancreatic malignancies promote M2-like polarization of TAMs and assist immune escape of tumor cells by regulating Gln metabolism reprogramming. Among them, PC regulates TAM polarization through the indirect axis of "Gln metabolic reprogramming-PSCs-immunity", while BTC regulates TAM polarization through the direct axis of "Gln metabolite-epigenetic". These differences are useful for the development of therapeutic strategies targeting tumor metabolism and immunity.

### 4.2. Tryptophan

Trp metabolic reprogramming is one of the key mechanisms of tumor immune escape and often significantly affects the TAM polarization by modulating the concentrations of key metabolites such as Kyn, 5-HT and indole. This mechanism is particularly active in biliopancreatic malignancies. In the Trp/Kyn metabolic pathway, tumor cells and TAMs express IDO1 highly, which metabolizes Trp to Kyn and other downstream products. Kyn can bind to the AhR of TAMs, resulting in nuclear translocation of AhR. Activation of AhR can reduce the polarization of M1-like TAMs and promote the polarization of M2-like TAMs^116^. For example, in esophageal squamous cells, Kyn can activate AhR to drive TAMs to acquire an immunosuppressive phenotype and induce the activation of M2-like TAMs through the activation of the AKT/glycogen synthase kinase-3β (GSK3β)/Il-8 signaling pathway, which can promote the proliferation and invasion of tumors^117^. At the same time, upregulation of IDO1 leads to a decrease in Trp concentration in the TME. Trp depletion activates general control nonderepressible 2 (GCN2). Activation of GCN2 triggers an integrated stress response, promotes polarization of M2-like TAMs by releasing cytokines such as IL-6 and IL-10, and directly inhibits the proliferation of CD8+ T cells, further enhancing tumor immune escape^118^. In addition, nicotinamide adenine dinucleotide (NAD), as an end product of the Trp/Kyn metabolic pathway, also promotes the polarization of M2-like TAMs^119^. Chitosanase 3-like protein 1 (YKL-40) is derived mainly from M2-like TAMs. It has been reported that it can activate the Trp/Kyn metabolic pathway through the upregulation of IDO1 and increase glioblastoma migration^120^. Wang Z *et al.*^22^ discovered that the knockdown of YKL-40 blocked the differentiation of M2-like TAMs and reprogrammed them into M1-like TAMs, thereby limiting tumor development. This recent study confirmed that YKL-40 was highly expressed in GBC and PC. Therefore, it is hypothesized that the occurrence and progression of biliary and pancreatic malignant tumors may also be inseparable from YKL-40-mediated reprogramming of Trp metabolism and polarization of M2-like TAMs. These studies suggest that the Trp/kyn metabolic pathway is an important reason for the activation of M2-like TAMs. In the Trp/5-HT metabolic pathway, Trp-derived 5-HT inhibits the LPS-induced release of proinflammatory factors, upregulates the expression of M2-like macrophages polarization-associated genes such as serine protease inhibitor 2 (SERPINB2), thrombospondin 1 (THBS1), stabilin 1 (STAB1) and collagen type XXIII alpha 1 chain (COL23A1), and decreases the expression of M1-like macrophages polarization-associated genes such as inhibin subunit beta A (INHBA), C-C motif chemokine receptor 2 (CCR2), MMP12 and plasminogen activator inhibitor-1 (PAI-1)^121^,^122^. It has been shown that 5-HT is required for follicular dedifferentiation and M2-like TAMs recruitment after inflammatory tissue injury in pancreatitis and is a key bridge between chronic inflammation and tumorigenesis^123^. In PDAC and CCA, this tumor-promoting mechanism is further amplified: tumor cells and CAFs express TPH1 and actively secrete 5-HT, which not only maintains the dedifferentiation and malignant proliferation of epithelial cells, but also accelerates the malignant progression of tumors by activating 5-HTR to recruit and polarize M2-like TAMs. Moreover, indoles metabolized from Trp are essential for mucosal barrier integrity and function in regulating AhR, T cells, DCs and macrophages. Riquelme E *et al.*^16^ found that lactic acid bacteria could promote the growth of PDAC by metabolizing Trp in food, producing indole, activating AhR on TAMs, inhibiting CD8+ T-cell infiltration within the tumor, and releasing IFN to kill tumor cells. Therefore, the accumulation of Trp metabolites in the TME is crucial for the polarization of M2-like TAMs and could serve as a predictive factor for immunotherapy in biliary and pancreatic malignant tumors. In summary, the accumulation of Trp metabolites in the TME is the core cause driving the polarization of M2-like TAMs and can be used as an important biomarker for predicting the efficacy of tumor immunotherapy.

### 4.3. Arginine

iNOS and ARG are key enzymes in the catabolism of Arg and exert a dual effect on driving the TAM phenotype and the progression of tumors to malignancy by competing for the substrate Arg and generating multiple metabolites. Rodriguez PC *et al.*^124^ observed no inhibition of tumor growth in mice lacking a functional immune system treated with ARG inhibitors. This result further confirmed that immune cells could assist in Arg metabolism reprogramming. Owing to the high demand for Arg uptake by tumor cells, the Arg content of the TME is low in the early stages of tumor development. In M1-like TAM, inflammatory mediators, in combination with iNOS, ASL and SLC7A2, upregulate ASS1 and increase Arg uptake, thereby increasing the concentration of iNOS-catalyzed substrates. iNOS catabolizes Arg into NO and citrulline. Citrulline can be regenerated into NO through the citrulline/NO cycle. NO has a dual effect on the progression of tumors to malignancy. A low concentration (<100 nmol/L) of NO inhibits the aggregation of immune cells, weakens the ability of tumor cells to undergo apoptosis, stimulates vascular formation and accelerates the progression of tumors to malignancy. However, high concentrations (400-1000 nmol/L) of NO accelerate tumor cell apoptosis and improve chemosensitivity^125^. With the continuous progression of tumors, the activity of iNOS gradually decreases, and the activity of ARG gradually increases under the action of cytokines secreted by Th2 cells. ARG, a characteristic enzyme of M2-like TAMs, can catalyze the hydrolysis of Arg into other metabolites, such as Orn and urea, which not only limits the accessibility of other antitumor immune cells to Arg and reduces the production of NO but also helps promote the proliferation of tumor cells, reduce the proliferation of T cells, and form the tumor immunosuppressive microenvironment^126^,^127^. A study revealed that Arg-treated TAMs presented increased numbers of M2-like TAMs and that the expression of CD163 in tumor tissues was positively correlated with the expression of Arg in the serum^128^. Orn derived from TAMs can also be converted to PAs, including putrescine, spermidine and spermine, by ODC. PAs can limit the activation of M1-like TAMs through chromatin modification and stimulate the expression of M2-like TAM-related genes^129^. Among them, spermine upregulates peroxisome proliferator-activated receptor γ expression through p53/thymine DNA glycosylase (TDG)-mediated DNA demethylation, which in turn promotes the polarization of M2-like TAMs. In addition, Arg can be decomposed into creatine under the catalysis of glycine transferase and guanidine acetate methyltransferase. Creatine has been shown to block the activity of iNOS by inhibiting the IFN-γ/JAK/STAT1 signaling pathway while promoting chromatin remodeling and activating the IL-4/STAT6 signaling pathway to regulate the expression of ARG, ultimately promoting M2-like TAM polarization^130^. However, maintenance of the creatine concentration in macrophages is achieved by SLC6A8. In other words, SLC6A8 has important clinical significance for the regulation of the TAMs phenotype and the associated immune response. It is believed that the infiltration of CD8+ T cells inhibits tumor development, while myeloid-derived suppressor cells and TAMs deplete extracellular Arg by producing a large amount of ARG1, thereby activating GCN2 and inhibiting the proliferation of CD8+ T cells^131^. In pancreaticobiliary malignancies, M2-like TAMs have a dual significance in Arg metabolic reprogramming. On one hand, Arg-dependent Biliary and pancreatic malignant tumor cells suffer from Arg depletion in the TME due to the upregulation of Arg metabolic reprogramming by TAMs, which leads to metabolic stress and forces the cells to initiate compensatory pathways. This further explains the involvement of KRAS-mediated Gln in synthesizing Orn in PC cells, in contrast to other tumor cells that rely on Arg to synthesize Orn. On the other hand, the long-term Arg depletion caused by TAM polarization may induce tumor cells to develop metabolic adaptability by upregulating transporters to enhance Arg uptake, intensifying the competitive metabolism of various cells for AAs in the TME. In PDAC and CCA, the highly fibrotic interstitium and the oncogenic background of chronic inflammation together shape the initial feature of TME: Arg depletion. Competitive consumption of Arg by tumor cells and TAMs led to a significant reduction in Arg content in the TME. At the same time, lactic acid produced by tumour cells, as a by-product of aerobic or anaerobic glycolysis, has a critical function in signalling, through inducing the expression of vascular endothelial growth factor and the M2-like polarization of TAMs^132^. This lactic acid derived from tumor cells is a potent inducer of ARG1 expression in TAMs. The lack of Arg content and the increase of lactic acid content jointly drive the reprogramming of Arg metabolic reprogramming in TAMs, showing that ARG1 expression is significantly up-regulated and iNOS expression is down-regulated, which ultimately promote the M2-like TAM polarization^133^. M2-like TAMs can not only exacerbate immune escape by secreting immunosuppressive cytokines such as IL-10 and TGF-β, but also promote collagen synthesis by metabolically generating products such as PAs and proline that directly stimulate tumor cell proliferation and activate CAFs. Ultimately, these effects exacerbate tumor fibrosis and disease progression, and more severe fibrosis in turn further worsens the Arg-depleting TME, creating a vicious cycle. Meanwhile, ARG1 has been reported to be a key driver of PC immunosuppression. Myeloid cells, represented by TAMs, are the main source of ARG1 in TME and correlate with patient survival^62^. In conclusion, Arg metabolic reprogramming plays a dual regulatory role on TAMs through iNOS and ARG pathways, but in immunosuppressive TME of biliary and pancreatic malignancies, ARG1-dominated tumor-promoting effects are dominant. Arg metabolic reprogramming is a central link in regulating the immune function of TAMs. Therefore, targeting the uptake, transport and metabolism of Arg, reversing the expression imbalance of iNOS/ARG1 and reshaping the metabolic pattern of TAMs have become important strategies to intervene in the polarization of TAMs and inhibit their tumor-promoting function.

### 4.4. Other AAs

In addition, the TME with high Met metabolic activity is often accompanied by T-cell dysfunction and increased infiltration of TAMs^134^. This property is particularly pronounced in biliopancreatic malignancies, whose highly fibrotic, immunosuppressed and nutrient-deprived TME provides the basis for Met metabolic reprogramming. Tumor cells and CAFs highly express Met transporters, which profoundly affect the polarization and immune effects of TAMs through competitive consumption of Met in the TME. Met can increase the ability of macrophages to secrete TNF-α and reduce the activity of ARG1^135^. In LPS-induced M1-like TAMs, the requirement for Met is derived from exogenous uptake. Met is used as a major methyl donor for SAM generation and subsequent methylation reactions, which are important for IL-1β production. Deficiency of Met inhibits IL-1β expression in LPS-induced M1-like TAMs^136^. In other words, Met metabolic reprogramming is involved in the formation of M1-like TAMs. Thus, it can be speculated that the high demand for Met by tumor cells leads to a decrease in the Met content in the TME, which inhibits the polarization of M1-like TAMs and weakens their antitumor effects. Meanwhile, Covarrubias AJ *et al.*^137^ conducted metabolic analysis of M2-like TAMs and found that Met metabolism is one of the pathways for their significant enrichment. In biliary and pancreatic malignancies, this enrichment has dual implications: on the one hand, TAMs need to enhance their Met metabolic reprogramming to adapt to low-nutrient, high-stress TME and maintain basic functions; On the other hand, this also makes the epigenetic status and polarization of TAMs more susceptible to fine regulation by Met and SAM availability. Moreover, It has been demonstrated that the inhibition of PHDGH or restriction of exogenous Ser uptake significantly increased the polarization of IFN-γ-activated macrophages and inhibited the polarization of IL-4-activated macrophages. Defective Ser metabolism increased insulin-like growth factor 1 (IGF1) expression by decreasing the SAM-dependent promoter abundance of H3K27me3. IGF1 then activated the p38-dependent JAK/STAT1 axis to promote M1-like TAM polarization and inhibited STAT6-mediated M2-like TAMs expression^138^. Remarkably, in Ser-deprived TME made by biliopancreatic malignancies, the polarization of both M1-like TAMs and M2-like TAMs is differentially regulated: while M1-like TAM polarization is severely impaired by metabolic insufficiency and epigenetic silencing, M2-like TAM polarization, although suppressed overall, may be selectively preserved for its key immunosuppressive functions. This combined effect leads to an eventual tendency for TAMs to undergo M2-like TAM polarization. Therefore, AA metabolic reprogramming has become an important target for AA depletion therapy. Especially in biliopancreatic malignancies, which are often driven by epigenetic modification mutations and exhibit metabolic deletion-type features, targeting AA metabolic reprogramming shows significant therapeutic potential and provides a new strategy for improving immunotherapy tolerance and reversing the immunosuppressive microenvironment. In addition, BCAA metabolic reprogramming is also actively involved in the regulation of TAMs biological functions and plays a vital role in the selective activation of TAMs. Lu M *et al.*^139^ found that the BCAA metabolic pathway is one of the metabolic pathways significant for M2-like TAM polarization. BCAAs can enter the TCA cycle as the carbon source and enhance mitochondrial oxidative phosphorylation, which is an important metabolic feature of M2-like TAMs. This implies that exogenous BCAAs promote M2-like TAM polarization. In contrast, knockdown of key transporters and metabolic enzymes such as SLC25A44, BCAT2, and branched-chain ketoacid dehydrogenase E1 (BCKDHA) inhibited M2-like TAMpolarization. It is worth noting that the BCAA metabolic reprogramming in TAMs is closely related to the expression of immune response gene 1 (IRG1) and the production of itaconic acid (ITA)^140^. IRG1 is significantly upregulated in PDAC and CCA. IRG1 overexpression inhibits M2-like TAM polarization and the progression of ICC to malignancy by inhibiting CCL18 to regulate STAT3 phosphorylation^141^. Meanwhile, it was shown that inhibition of BCAT1 expression reduced the production of ITA by macrophages through down-regulation of IRG1^142^. ITA can enter tumor cells and activate the nuclear factor erythroid-2 related factor 2 pathway in response to SLC13A3 transport, assisting tumor cells in resisting immunotherapy-induced ferroptosis^143^. In other words, targeting IRG1 by intervening in the BCAA metabolic reprogramming may reverse the immunosuppressive function of TAMs and enhance the efficacy of tumor immunotherapy. Although existing studies have confirmed the association between TAM polarization and BCAA metabolic reprogramming, the specific mechanisms of their roles in biliopancreatic malignancies still need to be further explored in depth. Finally, several studies have shown that taurine can balance the polarization state of macrophages and reduce inflammatory damage. Taurine inhibits LPS-induced and IFN-γ-induced M1-like TAMs and upregulates the expression of M2-like TAMs markers such as CD206, ARG1 and IL-10 through metabolic reprogramming mediated by the SAM/protein phosphatase 2Ac (PP2Ac) methylation/mitochondrial autophagy axis^144^.

In summary, AA metabolic reprogramming is closely associated with TAM polarization and related immune effects, emerging as a key factor regulating the function and activity of immune cells in biliary and pancreatic malignancies. Intervening in AA metabolic reprogramming may reshape the tumor-suppressive microenvironment, thereby enhancing immune cells' ability to recognize and eliminate tumor cells.

## 5. Tumor Therapy Targeting AA Metabolism Reprogrammining

**Figure 3:**
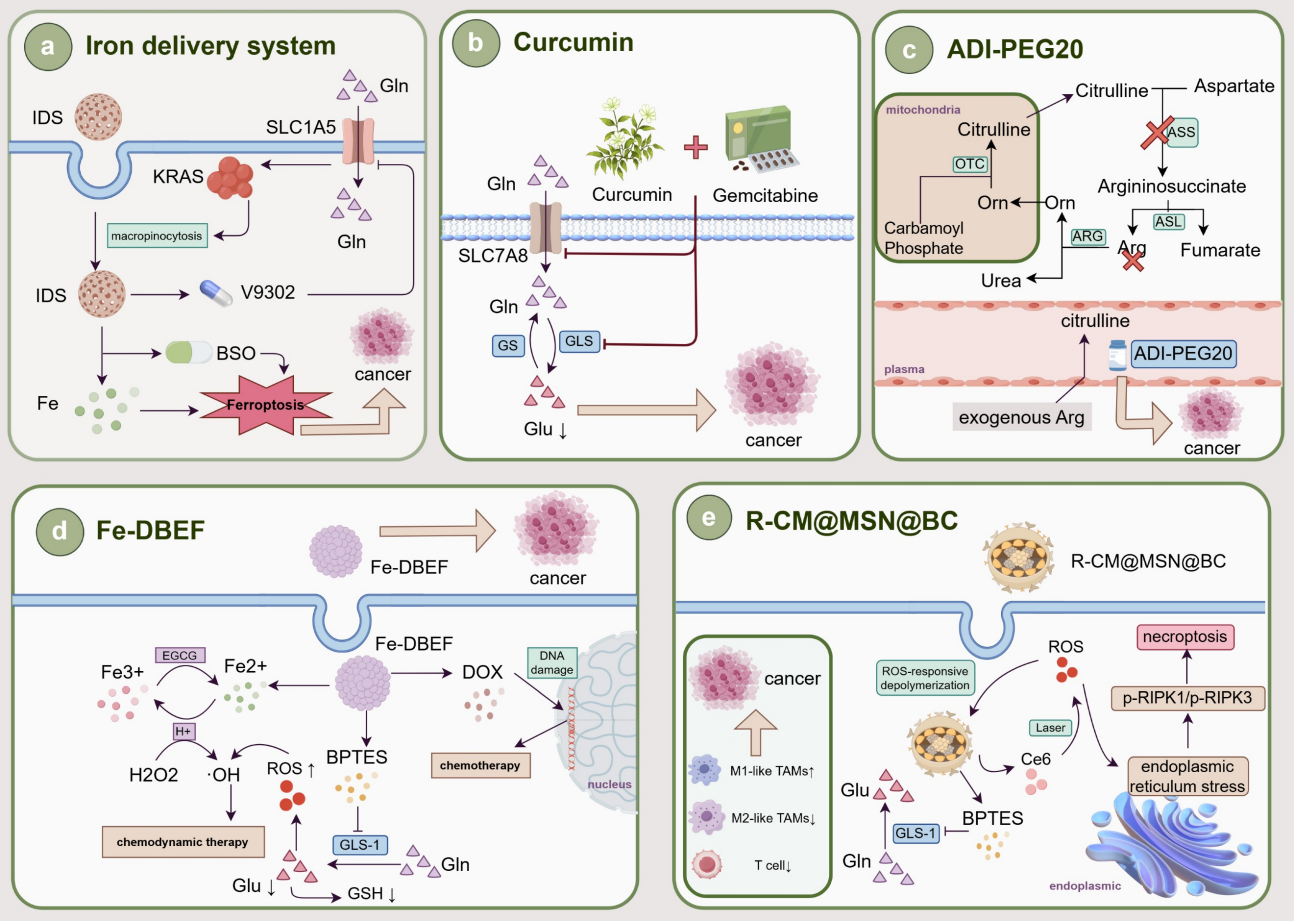
** Metabolic and immunoregulatory treatment strategies targeting AAs.** (a) A ferroptosis therapy with enhanced IDS endocytosis. The IDS loaded with V9302 enters the cells through endocytosis. The V9302 released by IDS can downregulate Gln metabolism reprogramming by inhibiting SLC1A5 and further stimulate the uptake of IDS by inducing KRAS-mediated macropinocytosis. The enhanced endocytosis resulting from the synergistic action of Gln and V9302 enables efficient delivery of iron and BSO to tumor cells, achieving tumor ferroptosis therapy. (b) Curcumin combined with GEM therapy. Curcumin can enhance the efficacy of GEM by downregulating the SLC7A8/Gln pathway. (c) Tumor starvation therapy. Normal cells synthesize Arg through the urea cycle, but many cancer cells lack ASS1 and rely on exogenous Arg to maintain metabolic energy. ADI-PEG 20 can decompose Arg in the blood, blocking the synthesis of proteins in tumor cells by downregulating Arg, while having no effect on normal cells. (d) A novel metal-polyphenolic-based multifunctional nanomedicine. This innovative formulation contained Pluronic F127-stabilized ferric ion-crosslinked EGCG nanoparticles loaded with the GLS-1 inhibitor BPTES and DOX. It can enhance ROS therapeutic sensitivity by decreasing GSH production in tumor cells, synergize with DOX to exacerbate DNA damage, promote apoptosis, and significantly inhibit tumor cell proliferation. (e) A biomimetic nano-system. R-CM@MSN@BC integrates mesoporous organosilicon nanoparticles with ROS-responsive diselenide bonds for controlled release of the Gln metabolism inhibitor BPTES and the photosensitizer Ce6. Upon laser irradiation, R-CM@MSN@BC executed both photodynamic and Gln-metabolic therapies, inducing necroptosis in tumor cells and triggering significant immunogenic cell death. R-CM@MSN@BC remodels the tumor immune microenvironment by increasing M1-like TAM polarization, reducing M2-like TAMs infiltration and T-cell depletion. (IDS: iron-delivery system; BSO: buthionine-sulfoximine; Gln: glutamine; Glu: glutamate; GLS: glutaminase; GS: glutamine synthetase; SLC: solute carrier; GSH: glutathione; ROS: reactive oxygen species; DNA: deoxyribonucleic acid; KRAS: Kirsten rat sarcoma viral oncogene homolog; IDH: isocitrate dehydrogenase; GEM: gemcitabine; Arg: Arginine; ASS: argininosuccinate synthase; ASL: argininosuccinate lyase; ARG: arginase; Orn: ornithine; ADI-PGE20: pegylated arginine deiminase; OTC: ornithine transcarbamylase; TAMs: tumor-associated macrophages; BPTES: bis-2-(5-phenylacetamino-1,3,4-thiadiazole-2-yl) ethyl sulfide; EGCG: epigallocatechin gallate; DOX: doxorubicin; Fe-DBEF: novel metal-polyphenolic-based multifunctional nanomedicine; RIPK: receptor interacting protein kinase).

AA metabolic reprogramming plays a central role in the development of biliopancreatic malignant tumors and the regulation of the immune microenvironment. Tumor cells compete with TAMs for the uptake of AAs in the TME, which not only directly supports malignant tumor proliferation, but also contributes to the polarization of M2-like TAMs and the formation of an immune-suppressive microenvironment that mediates therapeutic resistance. Therefore, targeting AA metabolic reprogramming has emerged as a dual strategy: both directly inhibiting tumor growth and reversing the immunosuppressive state. At present, a variety of drugs have been reported to exert tumor suppressive effects and increase chemotherapy efficacy by regulating AA metabolic reprogramming in biliary and pancreatic malignant tumors (Fig. 3). 6-diazo-5-oxo-L-nor-leucine (DON) is a potent Gln antagonist that has been evaluated in multiple clinical studies. However, DON lacks selectivity for tumor cells and induces intolerable gastrointestinal toxicity at therapeutic doses. Therefore, in-depth study of Gln metabolism inhibitors and expansion of their applications are crucial for tumor therapy. And hypoxia-activated prodrug of 6-diazo-5-oxo-L-nor-leucine (HDON) can be activated to DON by nitroreductase and esterase under hypoxic conditions, blocking tumor Gln metabolism, killing tumor cells, maintaining T cell activity, and enhancing the anti-tumor immune response^145^. HDON combined with combretastatin A4 nanoparticles achieves tumor-selective metabolic blockade. Sirpiglenastat (DRP-104), as a new generation of broad-spectrum Gln antagonist, has structurally improved the toxicity problem of DON, and is now in the clinical research stage. PDAC cells can compensate for the loss of Gln metabolism through the ERK signaling pathway. DRP-104 in combination with trametinib has been reported to inhibit the malignant proliferation of PDAC by compensating for the overexpression of the ERK signaling pathway^146^. Gln metabolic reprogramming is closely associated with ferroptosis. Chen Y *et al.*^147^ designed an iron-delivery system (IDS) with enhanced endocytosis for ferroptosis therapy. The IDS is characterized by Gln modification and can be recognized as a source of Gln nutrients for efficient endocytic uptake by PC cells. Due to the flexible binding properties of IDS to AA-like components, loading the Gln transporter inhibitor V9302 can further generate IDS with enhanced endocytosis. V9302 downregulates Gln by inhibiting SLC1A5, thereby inducing metabolic reprogramming in tumor cells. It enhances cellular uptake of Gln-modified IDS through KRAS-stimulated micropinocytosis. The enhanced endocytosis resulting from the synergism of Gln and V9302 enables the efficient delivery of iron and buthionine-sulfoximine (BSO) for ferroptosis tumor therapy, ultimately achieving ferroptosis-based tumor therapy. This research provides a novel approach to enhance intracellular drug delivery in tumors with heterogeneous nutrient metabolism by combining nutritionally modified nanomedicines with corresponding nutrient transporter inhibitors. Moreover, natural products such as parthenolide and curcumin show potential to inhibit CCA by modulating Gln metabolism reprogramming in preclinical studies^148^,^149^. Bis-2-(5-phenylacetamino-1,3,4-thiadiazole-2-yl) ethyl sulfide (BPTES), a GLS-1 inhibitor, in combination with the mTOR inhibitor everolimus, has demonstrated favorable efficacy in a mouse model of PDAC that lacks type II PI3K and PI3K-C2γ^150^. However, the poor water solubility, low metabolic stability and low bioavailability of BPTES hinder its clinical application.

Therefore, researchers conducted structural modifications and developed analogs such as CB-939 and IPN60090, which have already shown some clinical benefits^151^. At the same time, in order to improve the targeting delivery efficiency and therapeutic effects, various nano-carrier drugs have been successfully developed. Elgogary A^152^ used a proprietary emulsification process to encapsulate BPTES in nanoparticles. They found that although BPTES nanoparticles have a certain anti-tumor effect, some surviving tumor cells rely on glycolysis and glycogen synthesis. Therefore, they suggested that combined treatment with BPTES nanoparticles and metformin is more advantageous for targeting the metabolic heterogeneity of PC compared to the use of BPTES nanoparticles alone, and it may become a new treatment for PC. In addition, Dai Y *et al.*^153^ proposed an effective assembly strategy to synthesize a novel metal-polyphenolic-based multifunctional nanomedicine (Fe-DBEF). This innovative formulation contained Pluronic F127-stabilized ferric ion-crosslinked epigallocatechin gallate (EGCG) nanoparticles loaded with the GLS-1 inhibitor BPTES and the chemotherapy drug doxorubicin (DOX). It can enhance ROS therapeutic sensitivity by decreasing GSH production in tumor cells, synergize with DOX to exacerbate DNA damage, promote apoptosis, and significantly inhibit PC proliferation. Similarly, Zheng Q *et al.*^154^ developed a biomimetic nano-system designated R-CM@MSN@BC for immunotherapy and metabolic therapy of CCA. It could not only induce tumor cell necroptosis and trigger immunogenic cell death by controlling the Gln metabolism inhibitor BPTES, but also remodel the tumor immune microenvironment by increasing M1-like TAM polarization and reducing M2-like TAMs infiltration and T-cell depletion, thereby improving the effectiveness of anti- programmed cell death ligand 1 (PD-L1) immunotherapy. This strategy tightly integrates immunotherapy with Gln metabolism therapy and offers a practical and promising approach for the treatment of CCA. Furthermore, Arg plays a key role in tumor cell proliferation, survival and immune regulation. Currently, more and more studies have confirmed that interventions targeting Arg metabolic reprogramming pathways can effectively inhibit tumor malignant progression and remodel the tumor immune microenvironment. Arg starvation therapy can inhibit the migration, invasion and EMT of biliopancreatic malignancies^155^. And pegylated arginine deiminase (ADI-PGE20) effectively prevents the proliferation of CCA and PC cells by degrading Arg to citrulline, which has shown tolerable side effects in clinical trials^156^,^157^. This metabolic intervention strategy not only directly weakens the survival ability of tumor cells but also affects immune cell function by altering the AA metabolism balance in the TME. It is particularly noteworthy that Arg metabolism is closely related to the polarization state of TAMs. ARG1 and iNOS, as key enzymes in the process of Arg metabolic reprogramming, influence the polarization trend of TAMs. Attri KS *et al.*^158^ developed a real-time polymerase chain reaction-based assay in PC to monitor the transcriptional levels of ARG1 and iNOS to assess the status of TAM polarization under hypoxic conditions, which provided new ideas for immunotherapy and metabolic therapy. At the same time, there are some drugs that target other AA metabolic reprogramming. Telotristat ethyl (TE) is a TPH inhibitor that improves the frequency of bowel movements in patients with carcinoid syndrome by reducing the production of 5-HT. TE has a strong suppressive effect on CCA cells, which can increase the efficacy of chemotherapy, and is expected to improve the treatment of clinical CCA^159^. CH-223191 is a synthetic compound that antagonizes the Kyn/AhR signaling pathway in organisms, promotes M1-like TAM polarization, increases CD8+ T-cell infiltration, and improves the efficacy of programmed cell death 1 (PD-1)/PD-L1 immune checkpoint blockade therapy^160^. And methylthioadenosine phosphorylase (MTAP) is a key enzyme in the Met remediation pathway. MTAP deficiency leads to the accumulation of methylthioadenosine (MTA) in tumor cells, which in turn accelerates malignant tumor progression. AG-270, a highly active MAT2A inhibitor, has been reported to effectively inhibit the growth of a variety of MTAP-null tumor cells, including non-small cell lung cancer, PC and bladder cancer^161^. Furthermore, AAs can increase the expression of c-Myc by activating mTORC1, and upregulated c-Myc can act as an oncogene to regulate the transcription of SLCs. Therefore, there may be a positive feedback loop among c-Myc, SLCs and mTORC1. At present, SLC-targeted inhibitors have been reported to have broad tumor suppression effects on a variety of tumors^162^. Among them, JPH203, a potential new drug that targets SLC7A5, can significantly inhibit the progression of CCA to malignancy by blocking the uptake of Leu^83^. And Furuse J *et al.*^163^ demonstrated in a phase II study that JPH203 improved progression-free survival compared with placebo, with a safety profile in patients with advanced refractory BTC. In addition, the SLC7A5-specific PET probe can also serve as a companion diagnostic for SLC7A5-targeted therapy to select patients who are expected to benefit from the treatment. Recently, the cryo-electron microscopy structure of SLC7A5 has been resolved, which will aid in understanding the mechanisms of dynamic interactions between ligands and binding sites, and further design new compounds with higher activity. Moreover, sulfasalazine, an SLC7A11 inhibitor, has been shown to sensitize CCA cells to cisplatin. Sulfasalazine sensitizes CCA cells to cisplatin by killing CD44v9-positive cancer stem cells and altering the Trp catabolic pathway, thereby improving the drug resistance and chemotherapeutic efficacy of tumor cells^164^. In conclusion, AA metabolic reprogramming is strongly associated with the occurrence and progression of biliopancreatic malignancies, as well as the polarization of TAMs. AA metabolic reprogramming is gradually becoming an important target for metabolic therapy and immunotherapy for biliary and pancreatic malignancies (Table 3).

## 6. Perspectives

In summary, AA metabolic reprogramming, as a prominent component of tumor dysmetabolism, is clearly a key factor in the initiation and progression of biliary and pancreatic malignant tumors. AA enters cells via transporters and undergoes anabolic and catabolic processes catalyzed by associated metabolic enzymes. Compared to normal cells, malignant cells in biliary and pancreatic tumors often upregulate their capacity for AA metabolic reprogramming to support their progression toward malignancy. However, AA metabolic reprogramming is not a universal tumor phenomenon, exhibiting distinct tumor type specificity. For instance, while most studies indicate a high demand for Arg in tumor cells, and ASS1—the rate-limiting enzyme for Arg synthesis—is frequently overexpressed in CRC, biliary and pancreatic malignant tumors often rely more heavily on exogenous Arg uptake^165^. They achieve this by downregulating ASS1 to reduce endogenous Arg synthesis. Concurrently, compared to other malignancies that utilize Arg for Orn and PAs synthesis, CCA and PDAC cells rely more heavily on Gln as the precursor for Orn and PAs synthesis. This metabolic specificity is closely linked to the unique genetic background and TME of biliary and pancreatic malignant tumors. For PC cells, their AA metabolic reprogramming is closely associated with KRAS mutations and PSCs-mediated fibrosis. KRAS mutations drive Gln addiction by regulating Gln metabolism into aspartate and enhancing the NADPH/NADP+ ratio to maintain redox balance.

Meanwhile, PSCs not only supply BCAAs to support tumor proliferation but also overexpress ARG to drive Arg metabolic reprogramming, thereby fueling malignant progression. In contrast, AA metabolic reprogramming in BTC cells is more susceptible to IDH mutations and BAs. In CCA, mutated IDH induces Gln metabolism to produce D-2-HG, which induces DNA and histone hypermethylation, thereby driving tumor progression. Concurrently, BAs-induced oxidative stress drives Gln metabolism to synthesize GSH for oxidative stress resistance. Furthermore, compared to PC, Trp metabolic reprogramming in BTC favors the 5-HT pathway due to abnormal expression of TPH and MAO. These features not only distinguish BTC and PC from other malignancies in terms of AA metabolic reprogramming but also deepen our understanding of their metabolic specificity. Therefore, elucidating the unique AA metabolic reprogramming mechanisms in biliary and pancreatic malignant tumors is crucial.

In the development and progression of biliary and pancreatic malignant tumors, tumor cells and immune cells jointly shape an immunosuppressive TME. Tumor cells not only induce peripheral immune tolerance by releasing extracellular signals but also compete with immune cells for AAs, releasing downstream metabolites into the TME that directly target and impair immune cells. Similarly, dysregulation of AA metabolic reprogramming within immune cells also contributes to malignant tumor progression. The regulation of AA metabolic reprogramming by immune cells is complex and multifaceted. Current research indicates that GLS deficiency not only reduces the activation and proliferation of naive T cells and impairs Th17 cell differentiation but also promotes the differentiation and effector functions of Th1 cells and CD8+ T cells. Conversely, DCs can activate CD8+ T cells by upregulating Gln metabolic reprogramming. In other words, AA metabolic reprogramming exhibits a balanced regulatory effect on immune cells. As key innate myeloid cells, TAMs can polarize into distinct states between M1-like and M2-like TAMs under various signals released by AA metabolic reprogramming, profoundly influencing the functions of other immune cells and tumor progression. For instance, Trp catabolism not only produces Kyn that binds to TAM AhR to induce M2-like TAM polarization, but also activates GCN2 via Trp depletion to promote M2-like TAM polarization and suppress CD8+ T cell proliferation, further enhancing tumor cell immune evasion. Concurrently, TAMs can activate AA metabolic reprogramming by secreting related cytokines to promote malignant tumor progression. Compared to M1-like TAMs, M2-like TAMs dominate in biliary and pancreatic malignant tumors. TAM polarization is also closely associated with the characteristics of biliary and pancreatic malignant tumors. Within dense fibrotic microenvironments, activated PSCs secrete multiple cytokines that drive M2-like TAM polarization. In CCA and GBC, prolonged exposure to BAs and IDH mutations increases GSH and D-2-HG synthesis, enhancing M2-like TAM polarization. This marked enrichment of M2-like TAMs not only promotes tumor invasion and metastasis but also forms an “immune barrier” by enveloping tumor cells, thereby blocking the infiltration of effector immune cells and ultimately exacerbating immune evasion in biliary and pancreatic malignant tumors.

The pivotal role of AA metabolic reprogramming in biliary and pancreatic malignant tumors and their associated TAM polarization demonstrates AA's immense potential as a therapeutic target in cancer treatment. Modulating AA metabolic reprogramming may serve as an adjunctive approach to tumor immunotherapy. Currently, with ongoing research advancements, multiple therapeutic agents have entered preclinical and clinical trial phases. These agents exert antitumor effects and enhance immune function by regulating AA metabolic reprogramming in biliary and pancreatic malignancies. In preclinical studies, candidate drugs and therapeutic strategies have demonstrated diverse potential. For instance, IDS, Fe-DBEF and R-CM@MSN@BC combine nutritionally modified metabolic drugs with corresponding AA metabolic inhibitors. In tumors exhibiting heterogeneous nutritional metabolism, these agents not only enhance intracellular drug delivery but also regulate TAM polarization and other immune cell differentiation by blocking AA metabolic reprogramming. Concurrently, combination therapies integrating natural products like chrysanthemum lactones and curcumin with chemotherapeutic agents have demonstrated feasibility in preclinical models, laying groundwork for translational studies. At the clinical stage, compound development increasingly prioritizes balancing safety and efficacy. Although DON has a certain anticancer effect, clinical trials have shown that its therapeutic window is narrow and gastrointestinal side effects are significant. This is mainly because normal gastrointestinal cells also rely on Gln metabolic reprogramming, leading to the accumulation of DON in gastrointestinal tissues, causing harm to healthy tissues. DRP-104 addresses DON toxicity through structural optimization. Meanwhile, to address the treatment issues of metabolic compensation and metabolic heterogeneity in tumor cells, current research has found that the combination of BPTES and metformin can hinder the reliance of surviving tumor cells on glycolysis and glycogen synthesis. Moreover, Arg-deprivation therapies exemplified by ADI-PGE20 have demonstrated tolerable side effects. These studies indicate promising applications for AA metabolism-targeted therapies. However, although some drugs and immunotherapies have been proven to inhibit AA metabolism, reverse the imbalance of AA metabolism in the TME, and improve the immunosuppressive TME in biliopancreatic malignant tumor cells, owing to the wide variety of AAs and the fact that the treatment of certain metabolic targets has not been effectively developed, it is urgent to study the relationship among AA metabolic reprogramming, biliary and pancreatic malignancies and the related TAM polarization and discover more potential targets with clinical therapeutic value. Overall, with the continuous progress of research, the synergistic effect of targeted AA metabolic therapy and tumor immunotherapy has far-reaching clinical significance in inhibiting the progression of biliary and pancreatic tumors to malignancy.

## Figures and Tables

**Table 1:** The role of different kinds of AAs in the occurrence and development of biliary and pancreatic malignant tumors.

Type of AAs		Research	Ref.
Gln	PC	GLS is highly expressed in PDAC, and its succinylation modification promotes the malignant progression of tumors.	(35)
		PC cells take up Gln through the MUC5AC/β-catenin/c-Myc signaling pathway, which can accelerate tumor progression and drug resistance.	(39)
		Targeting pancreatic cancer Gln dependency confers vulnerability to GPX4-dependent ferroptosis.	(42)
		KRAS mutant PDAC increases the NADPH/NADP ratio through a specialized metabolic pathway of Gln to maintain cellular redox status.	(43)
		PSCs promote PC cell growth through Wnt/β-catenin/TCF7-mediated Gln metabolism.	(45)
		Yap/Myc signaling pathway induces PSCs activation by regulating Gln catabolism.	(46)
	CCA	The chemotherapy resistance of ICC patients is positively correlated with the expression of SLC1A5 and GLS-1.	(34)
		In CCA, platinum resistance is induced by high c-Myc levels through increased utilization of Gln.	(40)
		Gln supplementation regulates the ALK5/NOX1 axis to inhibit ferroptosis in ICC cells.	(41)
		IDH1/2 mutant CCA cells catalyze α-KG to produce D-2-HG to promote tumor progression.	(44)
	GBC	Lithobionic acid inhibits key enzymes of Gln metabolism and induces oxidative stress, triggering ferroptosis in GBC.	(48)
Trp	PC	NO/RUNX3/Kyn metabolic signaling enhances disease aggressiveness in PDAC.	(50)
		The serum expression level of 3-HAA and the ratio of 3-HAA/3-HK are inversely correlated with the risk of PC.	(51)
		5-HT and HTR2B agonists promote PC cell proliferation and inhibit apoptosis.	(57)
		The microbiota-derived Trp metabolite IAA promotes ROS accumulation, inhibits autophagy in PDAC cells and enhances chemotherapy efficacy.	(58)
	CCA	Compared with normal bile duct epithelial cells, the expression levels of TPH1, 5-HT1A, 5-HT2A/AB and 5-HT4/6 are elevated, and the expression level of MAO-A is decreased in CCA cells.	(55)
		High methylation of the promoter region of MAO-A attenuates the catabolic capacity of 5-HT and inhibits the growth of CCA cells.	(56)
	GBC	SAHA down-regulates the expression of IDO via inhibition of the JAK/STAT1 signaling pathway in GBC.	(52)
Arg	PC	p53 increases exogenous Arg uptake by upregulating SLC7A3 to drive the malignant progression of PDAC.	(61)
		Obesity can lead to a large accumulation of ammonia by upregulating ARG2, thereby promoting the malignant progression of PDAC.	(64)
		CAFs express ARG2, which synergistically promotes PDAC malignant progression with HIF-1α.	(65)
		KRAS-mutated PDAC induces OAT and polyamine synthase expression, increasing polyamine production and transport to sustain high growth rates.	(67)
		PRMT1 promotes epigenetic reprogramming associated with acquired chemoresistance in PC.	(70)
	CCA	ARG1 is an independent prognostic biomarker of ICC, and its expression level is positively associated with poor patient prognosis.	(63)
		PRMT5 and MEP50 are highly expressed in CCA and are closely related to methylation of Arg.	(69)
	GBC	gallbladder mucosa ODC activity is increased in patients with anomalous arrangement of the pancreaticobiliary duct, which may contribute to the pathogenesis of GBC.	(66)
Met	PC	Deletion of MSRA leads to the selective oxidation of Met residue M239 in PKM2, and thus accelerates metastasis of PDAC cells.	(73)
		Inactivation of p53 reduces SAM synthesis by regulating transcription of SLC43A2, making PC cells more susceptible to epigenetic interference under methylation stress.	(75)
	CCA	MAT1A expression is down-regulated in CCA and correlates with its promoter DNA hypermethylation.	(77)
		PHB1 is downregulated in CCA. PHB1 could positively regulate MAT1A and inhibit the expression of c-Myc, MAFG and c-MAF, ultimately promoting tumor progression.	(81)
	GBC	SAM blocks GBC cell proliferation and induces apoptosis by inhibiting the JAK2/STAT3 signaling pathway.	(82)
BCAAs	PC	A diet with a high content of BCAAs promotes the malignant progression of PDAC by stabilizing USP1-mediated BCAT2.	(89)
		In KRAS-mutated PDAC, Sky and TRIM21 accelerate tumor progression by regulating the ubiquitination modification of BCAT2 to maintain catabolism and mitochondrial respiration of BCAAs.	(90)
		CAFs exhibit exceptionally high BCAA catabolic capacity and generate BCKAs that contribute to malignant progression in PC cells.	(91)
	CCA	SLC7A5 knockdown attenuates the invasion and migration of CCA cells by upregulating microRNA-7 and inhibiting the 4F2hc signaling pathway.	(86)
		PPDPF is upregulated in CCA. It could block the interaction between MCCA and MCCB, inhibit Leu catabolism and activate mTORC1 signaling, thereby enhance the malignant phenotype of CCA cells.	(87)
Other AAs	PC	Cys depletion could upregulate ferroptosis by reducing the synthesis of GSH and CoA, and delay the malignant progression of PDAC.	(97)
		Mitochondrial calcium uniporter activates the Keap1/Nrf2 signaling pathway via a Cys-dependent mechanism, promoting PDAC progression and enhancing metabolic stress tolerance.	(98)
		Autophagy regulates Cys metabolism in PC cells by modulating SLC7A11. Loss of autophagy leads to intracellular Cys depletion.	(100)
		Neuronal peripheral axons release Ser to maintain the growth of PDAC cells in the absence of Ser/Gly.	(101)
	CCA	Redox-dependent modification of Cys and ferroptosis in bile might be mechanisms of ECC development.	(95)
		Downregulation of SLC7A11 in CCA cells inhibits the metabolic pathway of Cys. It leads to decreased GPX4 activity and lipid peroxide accumulation by reducing cystine and GSH synthesis, ultimately inducing ferroptosis and delaying tumor progression.	(96)
		PHGDH, a rate-limiting enzyme in the Ser/Gly metabolic pathway, is up-regulated in ICC associated with histone methyltransferase G9a.	(102)

**Table 2:** Relationship between AA metabolic reprogramming and macrophage polarization.

Type of AAs	Research	Ref.
Gln	GS maintains the formation of M2-like phenotype in TAMs.	(109)
	α-KG selectively activates M2-like macrophages by reducing the accumulation of H3K27me3 through the activation of JMJD3, and participates in fatty acid synthesis, assisting in the membrane reconstruction and paracrine of M2-like TAMs.	(110)
	α-KG inhibits the NF-κB signaling pathway, modulates IKKβ activity and attenuates the response of M1-like macrophages in a PHD-dependent manner.	(111)
	The Yap/Myc signaling pathway activates PSCs by regulating Gln metabolism, thereby driving PSCs to secrete multiple cytokines that induce M2-like TAM polarization.	(46,114)
	IDH1/2 mutations promote the conversion of Gln to D-2-HG. D-2-HG enhances SPP1 expression in fibroblasts, thereby inducing M2-like TAM polarization and impairing antitumor immunity.	(115)
Trp	Kyn bind to the AhR of TAMs, resulting in activation of AhR and promoting the polarization of M2-like TAMs.	(116)
	Trp depletion activates GCN2. GCN2 promotes M2-like TAM polarization by releasing cytokines such as IL-6 and IL-10, and directly inhibits CD8+ T cell proliferation.	(118)
	NAD is the end product of the Trp/Kyn metabolic pathway and promotes M2-like TAM polarization.	(119)
	YKL-40 is predominantly derived from M2-like TAMs, and it could activate the Trp/Kyn metabolic pathway by upregulating IDO1.	(120)
	5-HT inhibits release of pro-inflammatory factors, upregulates the expression of M2-like macrophages polarization-associated genes such as SERPINB2, THBS1, STAB1 and COL23A1, and decreases the expression of M1-like macrophages polarization-associated genes such as INHBA, CCR2, MMP12 and PAI-1.	(121-123)
	Lactic acid bacteria metabolize Trp to indole, which accelerates the polarization of M2-like TAMs by activating AhR.	(16)
Arg	TAMs uptake Arg and generate PAs, which promote the polarization of M2-like TAMs through p53/TDG-mediated DNA demethylation.	(129)
	Arg is decomposed into creatine, which could block the activity of iNOS by inhibiting the IFN-γ/JAK/STAT1 signaling pathway, while promoting chromatin remodeling and activating the IL-4/STAT6 signaling pathway to regulate the expression of ARG, ultimately promoting M2-like TAM polarization.	(130)
	Lactic acid produced by tumor cell metabolism is a potent inducer of ARG, promoting M2-like TAM polarization.	(133)
Other AAs	Met depletion inhibits IL-1β expression levels in LPS-induced M1-like TAMs.	(136)
	Inhibition of Met metabolism reduces the expression of H3K27me3, which promotes the polarization of M1-like TAMs by activating the JAK/STAT1 pathway, and reduce the polarization of M2-like TAMs by inhibiting IL-4 activation through the STAT6 pathway.	(138)
	Kockdown of SLC25A44, BCAT2 and BCKDHA inhibits M2-like TAM polarization.	(139)
	Taurine antagonizes M1-like TAM polarization by mitophagy-glycolysis switch blockage via dragging SAM-PP2Ac transmethylation.	(144)

**Table 3:** Tumor therapy targeting AA metabolic reprogramming.

Clinical treatment	Research	Ref.
HDON	HDON is activated under hypoxic conditions to exert anti-cancer effects. The combination of HDON with combretastatin A4 nanoparticles can achieve selective Gln metabolism blockade in tumors.	(145)
DRP-104	DRP-104 is a Gln antagonist that suppresses the malignant proliferation of PDAC by compensating for the overexpression of the ERK signaling pathway in combination with trametinib.	(146)
IDS	V9302 reduces Gln uptake by inhibiting SLC1A5 and enhances cellular uptake of Gln-modified IDS through RAS-stimulated macropinocytosis. The enhanced endocytosis resulting from the synergism of Gln and V9302 enables the efficient delivery of iron and BSO for ferroptosis tumor therapy.	(147)
Curcumin	curcumin could enhance the efficacy of GEM on CCA cells by downregulating the SLC7A8/Gln pathway.	(148)
Parthenolide	Parthenolide could inhibit the proliferation of CCA cells and promote apoptosis by interfering with Gln metabolism.	(149)
BPTES	The GLS-1 inhibitor BPTES hinders Gln activation and is often used in combination with everolimus to treat PDAC.	(150)
	The combination therapy of BPTES nanoparticles and metformin effectively targets tumor metabolic heterogeneity.	(152)
Fe-DBEF	Fe-DBEF reduces GSH generation and upregulates ROS by inhibiting GLS1. Excess ROS synergistically enhances the chemotherapy effect of DOX and effectively inhibits PC proliferation.	(153)
R-CM@MSN@BC	R-CM@MSN@BC inhibits BPTES and remodels the tumor immune microenvironment by increasing M1-like TAM polarization, reducing M2-like TAMs infiltration and T-cell depletion.	(154)
ADI-PGE20	ADI-PGE20 degrades Arg to citrulline and has tolerable side effects.	(156,157)
TE	TPH inhibitors TE can suppress CCA cells by reducing 5-HT synthesis and enhance the effectiveness of chemotherapy.	(159)
CH-223191	CH-223191 antagonizes the Kyn/AhR signaling pathway, promotes M1-like TAM polarization, increases CD8+ T cell infiltration, and enhances the efficacy of PD-1/PD-L1 immune checkpoint blockade therapy.	(160)
AG-270	MTAP deficiency leads to MTA accumulation within tumor cells, thereby accelerating malignant tumor progression. The MAT2A inhibitor AG-270 effectively suppresses the growth of various MTAP-deficient tumor cells.	(161)
JPH203	JPH203 inhibits the malignant progression of CCA by targeting SLC7A5 to block the uptake of Leu.	(83)
	JPH203 improves progression-free survival in patients with advanced refractory biliary tract cancer and is safe.	(163)
Sulfasalazine	Sulfasalazine sensitizes CCA cells to cisplatin by killing CD44v9-positive cancer stem cells and altering the Trp catabolic pathway, thereby improving drug resistance and chemotherapy efficacy of tumor cells.	(164)

## Abbreviations

BTC: biliary tract carcinoma
CCA: cholangiocarcinoma
PC: pancreatic cancer
GBC: gallbladder cancer
AA: amino acid
TME: tumor microenvironment
TAMs: tumor-associated macrophages
EAAs: essential amino acids
NEAAs: non-essential amino acids
Glu: glutamate
Gln: glutamine
GLS: glutaminase
mTOR: mammalian target of rapamycin
Ser: serine
GS: glutamine synthetase
BC: breast cancer
SLC: solute carrier
α-KG: α-ketoglutarate
TCA: tricarboxylic acid
GSH: glutathione
ROS: reactive oxygen species
GPX: glutathione peroxidase
DNA: deoxyribonucleic acid
ICC: intrahepatic cholangiocarcinoma
Myc: myelocytomatosis
c-Myc: cellular myelocytomatosis
MUC5AC: mucin 5AC
GEM: gemcitabine
ALK5: activin receptor-like kinase 5
NOX1: nicotinamide adenine dinucleotide phosphate oxidas1
KRAS: Kirsten rat sarcoma viral oncogene homolog
IDH: isocitrate dehydrogenase
D-2-HG: D-2-hydroxyglutarate
PSCs: pancreatic stellate cells
YAP: Yes-associated protein
BAs: bile acids
Trp: tryptophan
Kyn: kynurenine
5-HT: 5-hydroxytryptamine
TDO: tryptophan 2,3-dioxygenase
IDO: indoleamine 2,3-dioxygenase
AhR: aryl hydrocarbon receptor
3-HAA: 3-hydroxyanthranilic acid
3-HK: 3-hydroxykynurenine
IFN-γ: interferon-γ
DCs: dendritic cells
TPH: tryptophan hydroxylase
5-HTR: 5-hydroxytryptamine receptor
MAO: monoamine oxidase
RUNX3: runt-related transcription factor 3
NO: nitric oxide
iNOS: inducible nitric oxide synthase
IAA: indole-3-acetic acid
SAHA: suberoylanilide hydroxamic acid
Arg: arginine
ASS: argininosuccinate synthetase
ASL: argininosuccinate lyase
CRC: colorectal cancer
PDAC: pancreatic ductal adenocarcinoma
ARG: arginase
Orn: ornithine
ODC: ornithine decarboxylase
OAT: ornithine aminotransferase
HIF-1α: hypoxia inducible factor-1α
STAT: signal transducer and activator of transcription
PRMT: arginine methyltransferase
MEP50: methylated epitope protein 50
Met: methionine
MAT: methionine adenosyltransferase
SAM: S-adenosylmethionine
SAH: S-adenosylhomocysteine
Hcy: homocysteine
Cys: cysteine
MSRA: methionine sulfoxide reductase A
PKM2: pyruvate kinase M2
PHB1: prohibitin 1
HCC: hepatocellular carcinoma
MAFG: MAF bZIP transcription factor G
c-MAF: cellular musculoaponeurotic fibrosarcoma
Leu: leucine
BCAAs: branched-chain amino acids
CD: cluster of differentiation
mTORC1: mechanistic target of rapamycin complex 1
PPDPF: pancreatic progenitor cell differentiation and proliferation factor
MCC: 3-methylcrotonyl-CoA carboxylase
IL: interleukin
USP1: ubiquitin specific peptidase 1
BCAT: branched-chain amino acid aminotransferase
Syk: spleen tyrosine kinase
TRIM21: tripartite motif containing 21
BCKAs: branched-chain keto acids
Gly: glycine
ECC: extrahepatic cholangiocarcinoma
PHGDH: phosphoglycerate dehydrogenase
Keap1: Kelch-like ECH-associated protein 1
Nrf2: nuclear factor erythroid 2-related factor 2
TNF-α: tumor necrosis factor-α
LPS: lipopolysaccharides
Gas6: growth arrest-specific gene 6
CAFs: cancer-associated fibroblasts
EMT: epithelial-mesenchymal transition
NF-κB: nuclear factor kappa-B
HC-gp39: human cartilage glycoprotein-39
PI3K: phosphatidylinositol 3-kinase
AKT: protein kinase B
CCL: chemokine ligand
JAK: Janus kinase
H3K27me3: histone H3 lysine 27
JMJD3: Jumonji domain-containing protein 3
PHD: prolyl hydroxylase
IKKβ: inhibitor kappa B kinase β
TGF-β: transforming growth factor-β
SPP1: secreted phosphoprotein 1
GSK3β: glycogen synthase kinase-3β
NAD: nicotinamide adenine dinucleotide
YKL-40: chitosanase 3-like protein 1
SERPINB2: serine protease inhibitor 2
THBS1: thrombospondin 1
STAB1: stabilin 1
COL23A1: collagen type XXIII alpha 1 chain
INHBA: inhibin subunit beta A
CCR2: C-C motif chemokine receptor 2
PAI-1: plasminogen activator inhibitor-1
TDG: thymine DNA glycosylase
GCN2: general control nonderepressible 2
BCKDHA: branched-chain ketoacid dehydrogenase E1
IGF-1: insulin-like growth factor 1
IRG1: immune response gene 1
DON: 6-diazo-5-oxo-L-nor-leucine
ERK: extracellular regulated protein kinase
HDON: Hypoxia-activated prodrug of 6-diazo-5-oxo-L-nor-leucine
IDS: iron-delivery system
BSO: buthionine-sulfoximine
BPTES: bis-2-(5-phenylacetamino-1,3,4-thiadiazole-2-yl) ethyl sulfide
ADI: arginine deiminase
ADI-PGE20: pegylated arginine deiminase
TE: telotristat ethyl
PD-1: programmed cell death 1
PD-L1: programmed cell death ligand 1
EGCG: epigallocatechin gallate
DOX: doxorubicin
MTAP: methylthioadenosine phosphorylase
MTA: methylthioadenosine
DRP-104: sirpiglenastat
Fe-DBEF: novel metal-polyphenolic-based multifunctional nanomedicine
T helper cell: Th
CD8+T cells: cytotoxic T lymphocytes
TFEB: transcription factor EB
FNIP2: folliculin interacting protein 2
FLCN: folliculin
MMP: matrix metallopeptidase
Treg: regulatory T cell
NADPH: nicotinamide adenine dinucleotide phosphate hydrogen
NADP: nicotinamide adenine dinucleotide phosphate
